# Heterogeneity of Repolarization and Cell-Cell Variability of Cardiomyocyte Remodeling Within the Myocardial Infarction Border Zone Contribute to Arrhythmia Susceptibility

**DOI:** 10.1161/CIRCEP.122.011677

**Published:** 2023-05-02

**Authors:** Matthew Amoni, Dylan Vermoortele, Samaneh Ekhteraei-Tousi, Rosa Doñate Puertas, Guillaume Gilbert, Mohamad Youness, Bernard Thienpont, Rik Willems, H. Llewelyn Roderick, Piet Claus, Karin R. Sipido

**Affiliations:** 1 Department of Cardiovascular Sciences, Experimental Cardiology (M.A., S.E.-T., R.D.P., G.G., M.Y., R.W., H.L.R., K.R.S.), KU Leuven, Belgium.; 2 Imaging and Cardiovascular Dynamics, Department of Cardiovascular Sciences (D.V., P.C.), KU Leuven, Belgium.; 3 Laboratory for Functional Epigenetics, Department of Human Genetics (B.T.), KU Leuven, Belgium.; 4 Division of Cardiology, University Hospitals, Leuven, Belgium (M.A., R.W.).

**Keywords:** arrhythmias, cardiac remodeling, hypertrophy, magnetic resonance imaging, myocardial infarction, single-cell gene expression analysis, voltage-sensitive dye imaging

## Abstract

After myocardial infarction, the infarct border zone (BZ) is the dominant source of life-threatening arrhythmias, where fibrosis and abnormal repolarization create a substrate for reentry. We examined whether repolarization abnormalities are heterogeneous within the BZ in vivo and could be related to heterogeneous cardiomyocyte remodeling.

**Methods:**

Myocardial infarction was induced in domestic pigs by 120-minute ischemia followed by reperfusion. After 1 month, remodeling was assessed by magnetic resonance imaging, and electroanatomical mapping was performed to determine the spatial distribution of activation-recovery intervals. Cardiomyocytes were isolated and tissue samples collected from the BZ and remote regions. Optical recording allowed assessment of action potential duration (di-8-ANEPPS, stimulation at 1 Hz, 37 °C) of large cardiomyocyte populations while gene expression in cardiomyocytes was determined by single nuclear RNA sequencing.

**Results:**

In vivo, activation-recovery intervals in the BZ tended to be longer than in remote with increased spatial heterogeneity evidenced by a greater local SD (3.5±1.3 ms versus remote: 2.0±0.5 ms, *P*=0.036, n_pigs_=5). Increased activation-recovery interval heterogeneity correlated with enhanced arrhythmia susceptibility. Cellular population studies (n_cells_=635–862 cells per region) demonstrated greater heterogeneity of action potential duration in the BZ (SD, 105.9±17.0 ms versus remote: 73.9±8.6 ms; *P*=0.001; n_pigs_=6), which correlated with heterogeneity of activation-recovery interval in vivo. Cell-cell gene expression heterogeneity in the BZ was evidenced by increased Euclidean distances between nuclei of the BZ (12.1 [9.2–15.0] versus 10.6 [7.5–11.6] in remote; *P*<0.0001). Differentially expressed genes characterizing BZ cardiomyocyte remodeling included hypertrophy-related and ion channel–related genes with high cell-cell variability of expression. These gene expression changes were driven by stress-responsive TFs (transcription factors). In addition, heterogeneity of left ventricular wall thickness was greater in the BZ than in remote.

**Conclusions:**

Heterogeneous cardiomyocyte remodeling in the BZ is driven by uniquely altered gene expression, related to heterogeneity in the local microenvironment, and translates to heterogeneous repolarization and arrhythmia vulnerability in vivo.

**What is Known?**

- The infarct and its surrounding border zone (BZ) are the source of arrhythmias after myocardial infarction.
- Structural remodeling with interspersion of fibrosis and cardiomyocytes creates a substrate for reentry to occur.
- Altered repolarization of cardiomyocytes after myocardial infarction in the BZ likely contributes to this substrate.

**What the Study Adds**

- Altered repolarization in the BZ is not homogeneous. Within the BZ of a reperfused myocardial infarction, studied in the pig, there is cell-to-cell variability of cardiomyocyte remodeling, with phenotypic heterogeneity of repolarization and hypertrophy.
- Heterogeneous cardiomyocyte remodeling is driven by highly variable gene expression including genes related to hypertrophy (*NPPB* and *INPP5F*) and action potential duration (*KCNT2* and *SCN3B*). The upstream transcription factors are consistent with variable signaling pathways, such as the documented variable wall stress within the BZ.
- This heterogeneous cellular remodeling translates to local heterogeneity of repolarization in vivo within the BZ that contributes to postmyocardial infarction arrhythmia vulnerability.

Ventricular arrhythmias and sudden death are major complications after myocardial infarction (MI), with a particularly high incidence in the first months after the acute event.^1^ The transition zone between the scar and healthy myocardium is the most vulnerable region and a prime target for ablation therapy.^2,3^ In this border zone (BZ), the mix of surviving cardiomyocytes and fibrous tissue forms a perfect substrate for reentry. Whether BZ cardiomyocytes also undergo electrical remodeling that enhances the reentry, distinct from the overall remodeling in the post-MI heart, is likely, but data are more scarce, in particular with regard to heterogeneity within the BZ.

Many studies to identify mechanisms linking MI and arrhythmias have used small animal models, particularly rats and mice, and reported on cardiomyocytes from the viable left ventricle (LV).^4–7^ These studies predominantly used a permanent coronary ligation model, not representative of current clinical practice.^8^ Studies in the dog^9^ and pig^10^ have identified regional action potential (AP) changes critical for arrhythmogenesis including Purkinje cell and cardiomyocyte remodeling in the BZ. These studies exploited the advantage that large animal models may be more suitable to identify this vulnerable BZ region within the post-MI heart, as well as to connect in vivo and ex vivo findings.^11^ In the pig with 5-month chronic microvessel occlusion distal to the first diagonal branch of the left anterior descending coronary artery, an AP shortening in cardiomyocytes from the BZ compared with a lengthening in the remote region was reported.^12^ We studied regional AP changes 6 weeks after MI without reperfusion and only found differences between regions during adrenergic stimulation.^13^

In current clinical practice, reperfusion is established as soon as possible after acute coronary occlusion. This intervention salvages myocardium but also subjects the BZ to intense remodeling driven by local inflammation.^14^ It is conceivable that local differences in perfusion and inflammation lead to further heterogeneity within the BZ, consistent with clinical observations of areas of greater vulnerability within the BZ.^2^ In the pig with an MI following ischemia and reperfusion, adrenergic stimulation also uncovered regional vulnerability and heterogeneity, with clusters of premature ventricular beats dispersed within the BZ.^15^ Such heterogeneity could result from the different ultrastructure, whether related to fibrosis or innervation, but could also reflect a diversity in cardiomyocyte populations.

The aim of the present study is to uncover potential heterogeneity within the BZ, at 4 weeks after MI, a time of high arrhythmic risk.^1^ Our focus is on the repolarization process, which is a major factor in facilitating reentry,^16^ with heterogeneity of repolarization a feature of VT circuits after MI.^17^ We use a combination of novel and complementary approaches reaching across in vivo electrophysiology, cell function in large populations,^18^ and gene expression in cardiomyocyte subpopulations.

## Methods

Detailed methods are provided in the Supplemental Material, including the supporting references. The data and analytical methods supporting the findings of this study are available from the corresponding author upon reasonable request. RNA sequencing data will be made available on completion of a follow up study.

### Preclinical Animal Model of MI

Animal handling was in accordance with the National Institutes of Health Guide for the Care and Use of Laboratory Animals and the EU Directive for animal research 2010/63/EU. Experimental protocols were approved by the KU Leuven Animal Ethics Committee (ECD137/2018). The experimental workflow is illustrated in Figure S1. All animal procedures were performed under general anesthesia. MI was induced in 22 domestic pigs (TN70 strain; weight, 30–35 kg; Topigs Norsvin) by 120-minute balloon occlusion of the left anterior descending coronary artery followed by reperfusion, while 11 pigs underwent a Sham procedure.^15^ Two MI animals experienced sudden death within 1 week after MI.

### Cardiac Magnetic Resonance Imaging

After 1 month, animals underwent cardiac magnetic resonance imaging. The LV structural and functional parameters are summarized in Table S1. The BZ was defined as the region with signal intensity 20% to 70% of the maximum intensity (infarct core) and the remote region as the posterior wall opposite to the BZ.

### In Vivo Cardiac Electrophysiology

Electroanatomical mapping of the LV was performed as described previously.^15^ Contact mapping was performed to generate the LV map paired to the EnSite multielectrode array and define the BZ as points with bipolar voltage >0.5 and <1.5 mV. After ≈20-minute stabilization, noncontact mapping using the multielectrode array was performed to record 2048 noncontact local electrograms for 5 minutes.

Mapping data were processed to calculate the local activation-recovery interval (ARI) for each local electrogram and reconstruct a spatially dense map using the E-field method.^19^

After 1 to 3 days of recovery, animals were euthanized for tissue harvesting and cellular experiments.

### Isolated Cardiomyocyte Electrophysiology and Phenotyping

Cardiomyocytes were isolated from the anterior and septal MI BZ, from the remote region and from similar regions in Sham^15^; each animal thus contributed matched data from the diffferent regions. Freshly isolated cardiomyocytes were studied using whole-cell patch-clamp mode.^13^ For optical recording of APs, cells were loaded with di-8ANEPPS and randomly imaged on the stage of an inverted microscope (Nikon TiE, Germany), with the investigator blinded to the region. Cells were superfused with Tyrode solution at 37 °C and field stimulated at 1 Hz. AP duration (APD) was defined at 25% (APD25), 50% (APD50), and 90% repolarization (APD90).

### Single-Nucleus RNA Sequencing and Gene Expression Analysis

#### Isolation of Cardiac Nuclei

Tissue samples collected under RNase free conditions from the BZ and remote regions were immediately snap frozen in liquid nitrogen and stored at −80 °C. Debris-free suspensions of intact nuclei were generated as described in the Supplemental Material.

#### 10× RNA Single-Nucleus RNA Sequencing and Analysis

Single-nucleus RNA-seq libraries were prepared on ≈6000 nuclei per sample (10× genomics) and sequenced at a depth of ≈30 000 reads per nucleus. Transcriptome data sets per sample were provided by CellRanger V5, followed by removal of ambient RNA and low complexity libraries. Sample data sets were merged, further processed, and cell-type clusters generated using Seurat V4. Clusters were visualized using a uniform manifold approximation and projection approach and were annotated based on their expression of established cell type–specific markers.^20^

Variability of gene expression was analyzed at the cellular level, using the mean of Euclidean distance of each cell relative to other cells in each region, and at the level of gene expression, assessing variability using BASiCS. Expressed/dispersed genes were identified with an expected false discovery rate <0.10. Regulatory elements and involved TFs (transcription factors) were identified using the RcisTarget (version 1.14.0) tool from SCENIC, an algorithm that prioritizes chromatin immunoprecipitation-validated TF binding to given targets.^21^

### Data and Statistical Analysis

Offline data analysis was performed blinded where feasible. Data are presented as mean±SD, unless otherwise stated. All statistical analyses and sample numbers (n_pigs_ and n_cells_) are indicated in the figure legends.

## Results

### Variable Repolarization in the BZ In Vivo

**Figure 1.**
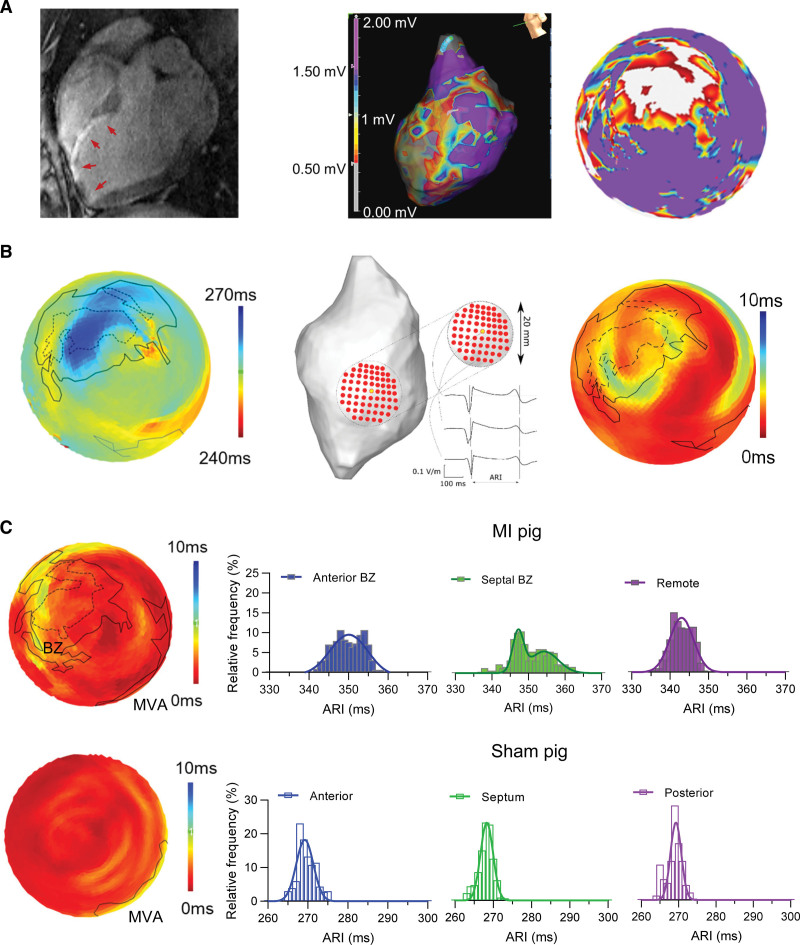
**Regional activation-recovery intervals (ARIs) in vivo after myocardial infarction (MI). A**, Example of cardiac magnetic resonance imaging (**left**) with corresponding electroanatomical map (**middle**) and contact voltage polar map (**right**) illustrating the extent of infarct (infarct size, 18.2%; ejection fraction, 46.8%), and the electroanatomical border zone (BZ) defined as bipolar voltage between 0.5 and 1.5 mV, with the white area the infarct zone. **B**, **Left**, Noncontact electrogram ARI map calculated with the E-field method. **Middle**, Local ARI heterogeneity, the spatial repolarization heterogeneity (around the yellow center point) is defined as SD of ARIs within the 1-cm radius. **Right**, Polar map of ARI heterogeneity. The outer edge of the infarct and border zone are annotated on the polar maps using dotted and full lines, respectively. **C**, Examples of polar maps of the local ARI heterogeneity of the left ventricle (LV; **left**) and corresponding regional distribution of ARIs (**right**) in MI (**top**) and Sham (**bottom**). MVA indicates mitral valve annulus.

We assessed the presence of regional variability of repolarization in vivo. Using a recently developed algorithm, we mapped the local repolarization time from spatially dense electrograms of noncontact electroanatomical maps of the LV.^19^ Contact mapping defined the infarct, BZ, and remote regions, which were translated to polar maps to aid visualization (Figure 1A). The local ARIs, as a measure of repolarization time, were plotted on the polar map (Figure 1B, left). Heterogeneity of repolarization measured as the SD of ARI values in a region of 1-cm radius around each endocardial point (Figure 1B, middle) was visualized using the polar map (Figure 1B, right). Three-dimensional electroanatomical maps, ARI maps, and heterogeneity maps from different views are shown in Figure S2.

Figure 1C shows an example of the ARI heterogeneity polar map and corresponding distribution of ARIs from an MI and Sham animal. The distribution of ARIs from the MI BZ had a wider profile compared with the remote region, consistent with the visual image of dispersion. Such a profile, indicative of heterogeneity of repolarization, was not seen in the histograms of the ARIs of the remote region or in Sham.

**Figure 2.**
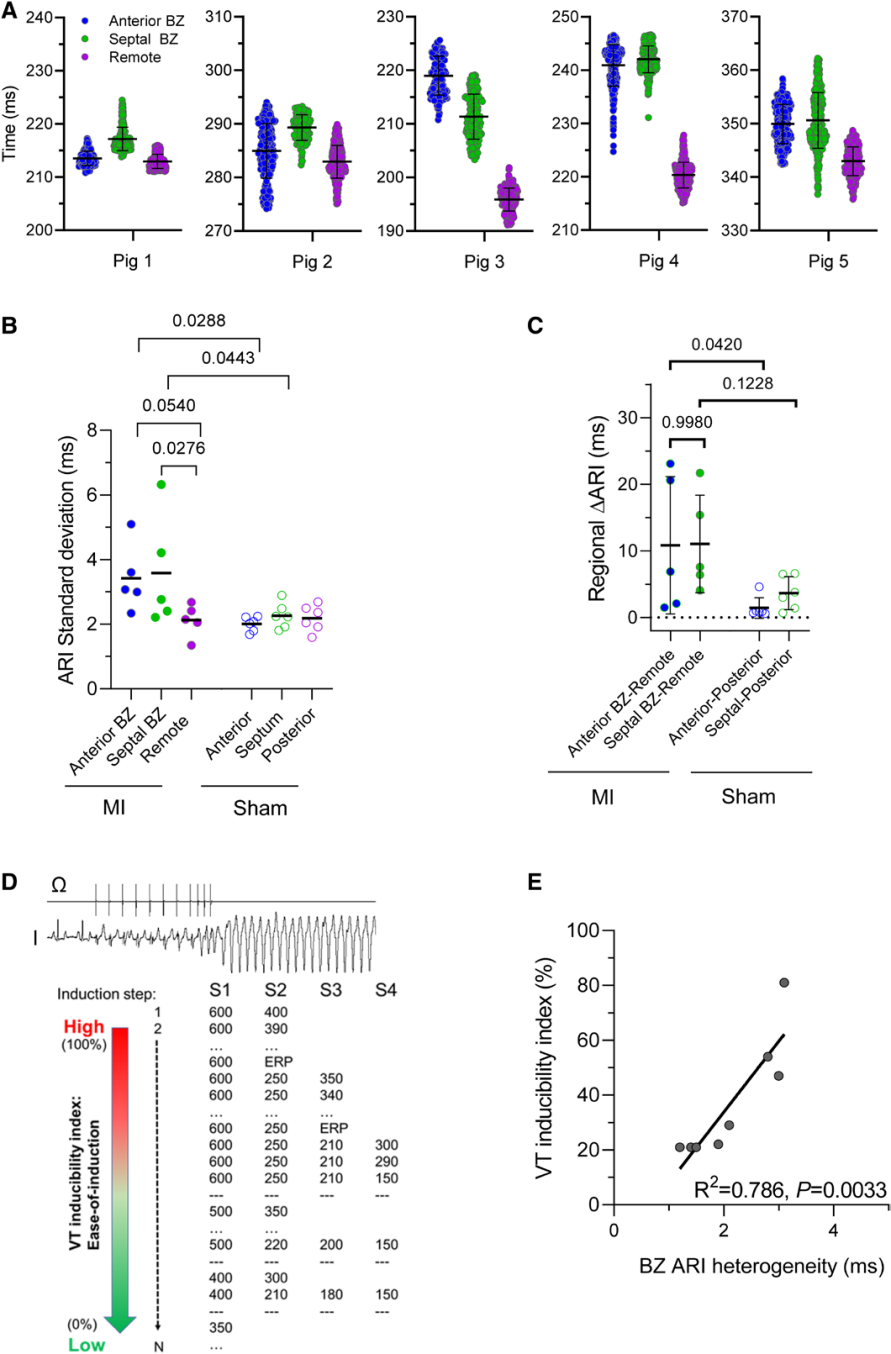
**Quantification of in vivo heterogeneity of repolarization. A**, Regional in vivo activation-recovery intervals (ARIs) from myocardial infarction (MI). Pooled data on ARIs of the different regions in vivo for each MI (n_pigs_=5, n_ARIs_=112–468 per region). **B**, Regional ARI heterogeneity. Summary data of ARI heterogeneity quantified by regional SD per pig from **A**. Mixed-effects model ANOVA with Bonferroni post test. **C**, Regional heterogeneity difference. Regional differences in ARI dispersion quantified by pig as mean ARI differences. Mixed-effects model ANOVA with Bonferroni post test. **D**, Illustration of ventricular tachycardia induction protocol and the scale to quantify ease of induction. **E**, Arrhythmogenicity correlation. Correlation of in vivo border zone (BZ) heterogeneity and arrhythmia inducibility in MI. n=8. Linear regression analysis.

A similar pattern of ARIs was seen in all MI pigs. Figure 2A shows ARI values for anterior and posterior BZ and the remote region for each pig (Sham data are presented in Figure S3). The visual impression of greater heterogeneity of local ARIs in the BZ was quantified as the SD for each pig. These values were significantly larger in the BZ (Figure 2B). Since in vivo heart rate influences APD, and it is not identical between different pigs, pooling the ARI data was not appropriate. Therefore, we used the magnitude of the regional difference between BZ and remote for each animal to assess mean ARI differences between regions across the group of animals (Figure 2C). These data suggest that the repolarization in the BZ was both more heterogeneous and overall longer than that of the remote region.

The observation of local ARI heterogeneity in the BZ could contribute to a functional substrate for arrhythmias. To investigate the relationship between ARI heterogeneity and arrhythmogenicity, we performed VT induction in a group of 8 MI pigs. We quantified arrhythmogenicity as the ease of induction during a standard stepwise programmed stimulation protocol introducing up to 3 extra stimuli (Figure 2D). The degree of BZ ARI heterogeneity was strongly correlated with the VT inducibility index (Figure 2E), suggesting that indeed heterogeneity of repolarization within the BZ contributes to arrhythmogenesis in vivo.

### Heterogeneity of Repolarization Within the Population of BZ Myocytes

**Figure 3.**
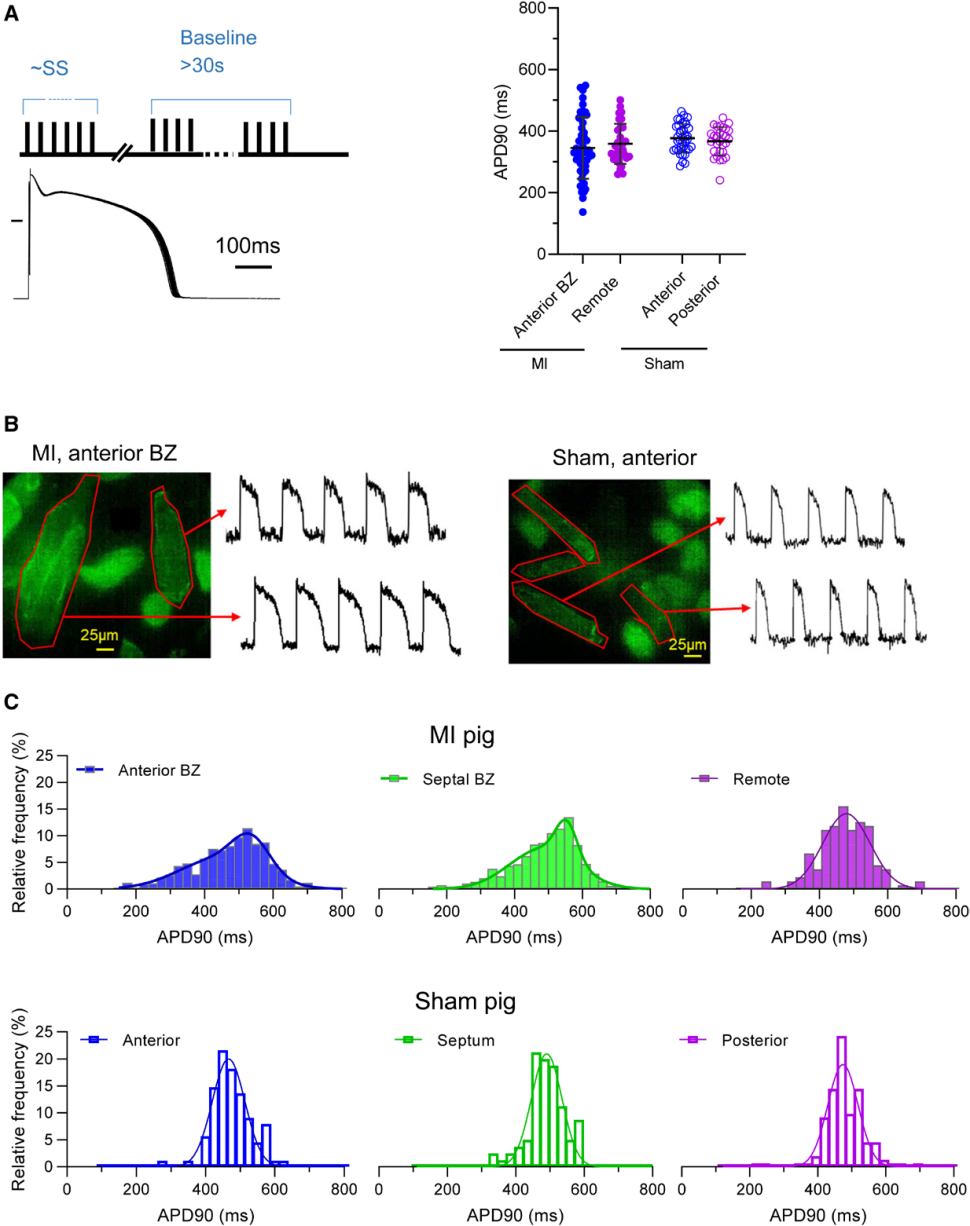
**Action potential duration at 90% repolarization (APD90) of isolated myocytes from different regions. A**, **Left**, Protocol and representative example of 30 steady-state action potentials recorded under patch-clamp and averaged for analysis. **Right**, Summary of regional cellular APD90 profiles from myocardial infarction (MI; n_pigs_=7, n_cells_=4–9 per region) and Sham (n_pigs_=6, n_cells_=4–8 per region). Multilevel mixed-model ANOVA with Bonferroni correction. **B**, Examples of border zone (BZ; **left**) and Sham (**right**) cells loaded with the voltage dye di-8ANEPPS and corresponding action potentials recorded. Scale bar, 25 µm. **C**, Examples of the distribution of cellular APD90 of populations of isolated cells from each region of an MI (n_cells_=212–312 per region) and Sham pig (n_cells_=133–254 per region) illustrating increased heterogeneity in the MI BZ.

To examine whether heterogeneity in cardiomyocyte remodeling underlies this heterogeneity of repolarization in the BZ, we studied repolarization in cardiomyocytes isolated from the anterior and septal BZ, the remote region, and similar regions in Sham. In a first set of experiments, we recorded cellular APs using conventional whole-cell patch-clamp recording. Figure 3A, left, illustrates the stimulation protocol; the right panel shows the summary data of all cells. In this substantial data set of 7 MI and 6 Sham animals, each pig contributing 4 to 9 cells per region (n_cells_=149), no significant difference in APD between regions was detected. However, it was evident that the APD of BZ cardiomyocytes had a larger range than that of the remote and matched Sham regions of equivalent sample size. This difference between BZ and other regions could reflect an interindividual variability or an intraregional variability and, by extension, obscure differences in mean values.

To probe the existence and nature of intraregional heterogeneity within each animal, we implemented an optical method using a voltage-sensitive dye to record APs from 200 to 400 cardiomyocytes/pig, in an unbiased manner (Figure 3B). The APs of 10 tracings at the end of a 15-s recording were averaged, and APD was measured. Figure 3C illustrates the distribution of cellular APDs in a representative MI and Sham pig. Visually, the BZ distributions were wider and skewed toward a longer APD, in both anterior and septal BZ, which was not seen in the remote region or in Sham animals.

**Figure 4.**
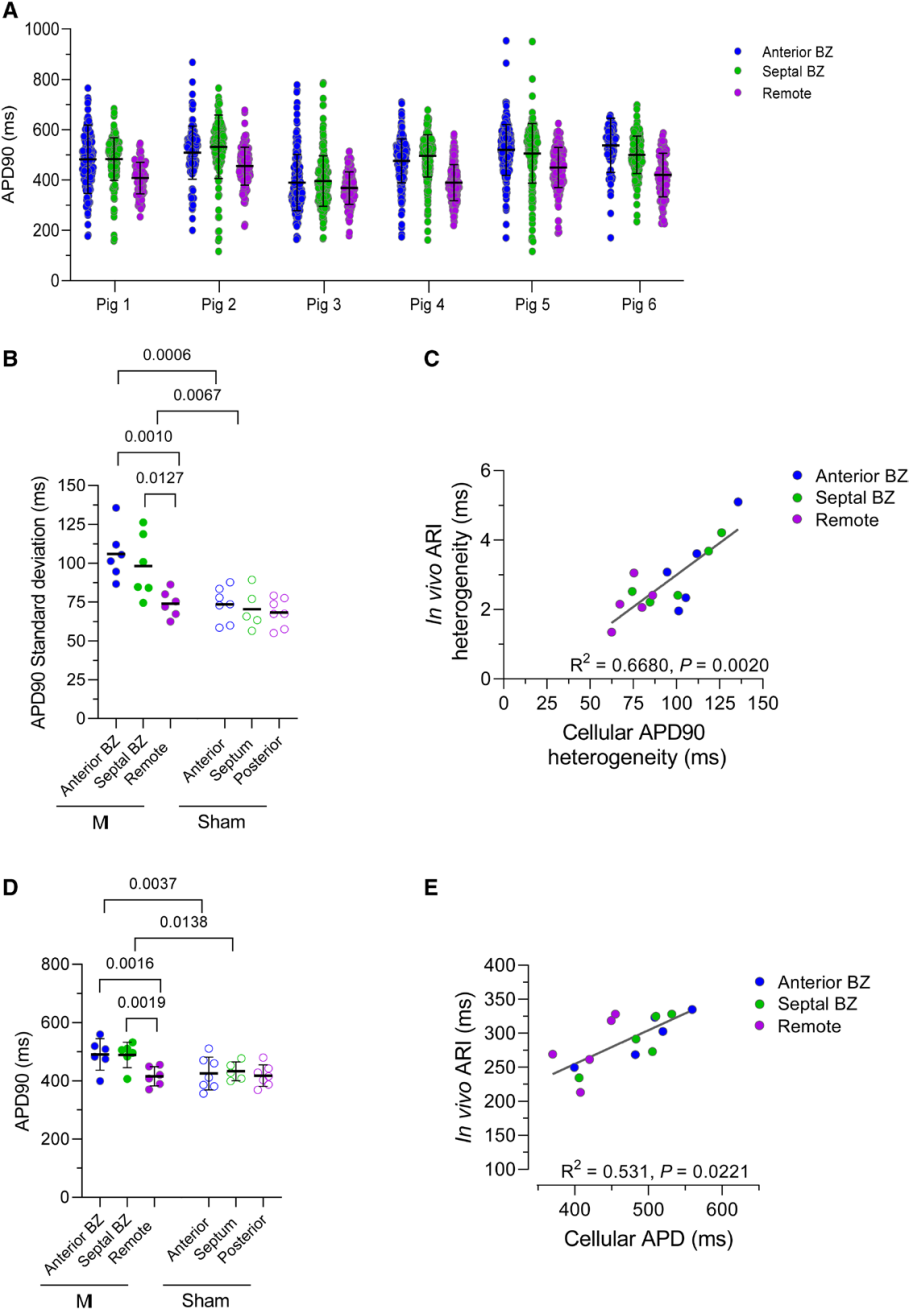
**Quantification of regional cellular action potential duration heterogeneity. A**, Regional cellular action potential duration at 90% repolarization (APD90) in each myocardial infarction (MI) animal, n_pigs_=6 (n_cells_=86–216 per region). **B**, Regional APD90 heterogeneity. Summary of cellular APD90 heterogeneity as SD per pig, mixed-model ANOVA with Bonferroni post test. **C**, Correlation of regional cellular and in vivo heterogeneity in MI (n_pigs_=5), linear regression analysis. **D**, Regional repolarization duration. Mean data of cellular APD90 pooled by pig from **A**, mixed-model ANOVA with Bonferroni post test. **E**, Correlation of regional cellular and in vivo repolarization duration in MI (n_pigs_=5), linear regression analysis.

Figure 4A represents the complete data set of 6 MI (data set of 7 Sham animals is presented in Figure S4A), illustrating the large spread of cellular APD in MI BZ. Moreover, the effect was apparent at both the anterior and septal subregions. Intraregion heterogeneity of cellular APDs was quantified by the SD in each region and animal and was larger in BZ compared with remote (Figure 4B). These data are in line with our in vivo findings; that is, we observe heterogeneity of repolarization in the BZ, without distinctive features between septal or anterior BZ, but not in the remote region. Furthermore, there was a strong correlation between in vivo and cellular heterogeneity (Figure 4C).

From this large data set of optically derived APs, we could detect an average increase in APD in the BZ compared with remote and without difference between anterior and septal subpopulations (Figure 4D; APD50 and APD25 data in Figure S4B). The mean regional APD correlated only moderately with the mean of the regional in vivo ARI (Figure 4E), possibly related to the differences in heart rate in vivo versus stimulation of 1 Hz in cells.

### Heterogeneous Myocyte Hypertrophy in BZ and Remote Regions

**Figure 5.**
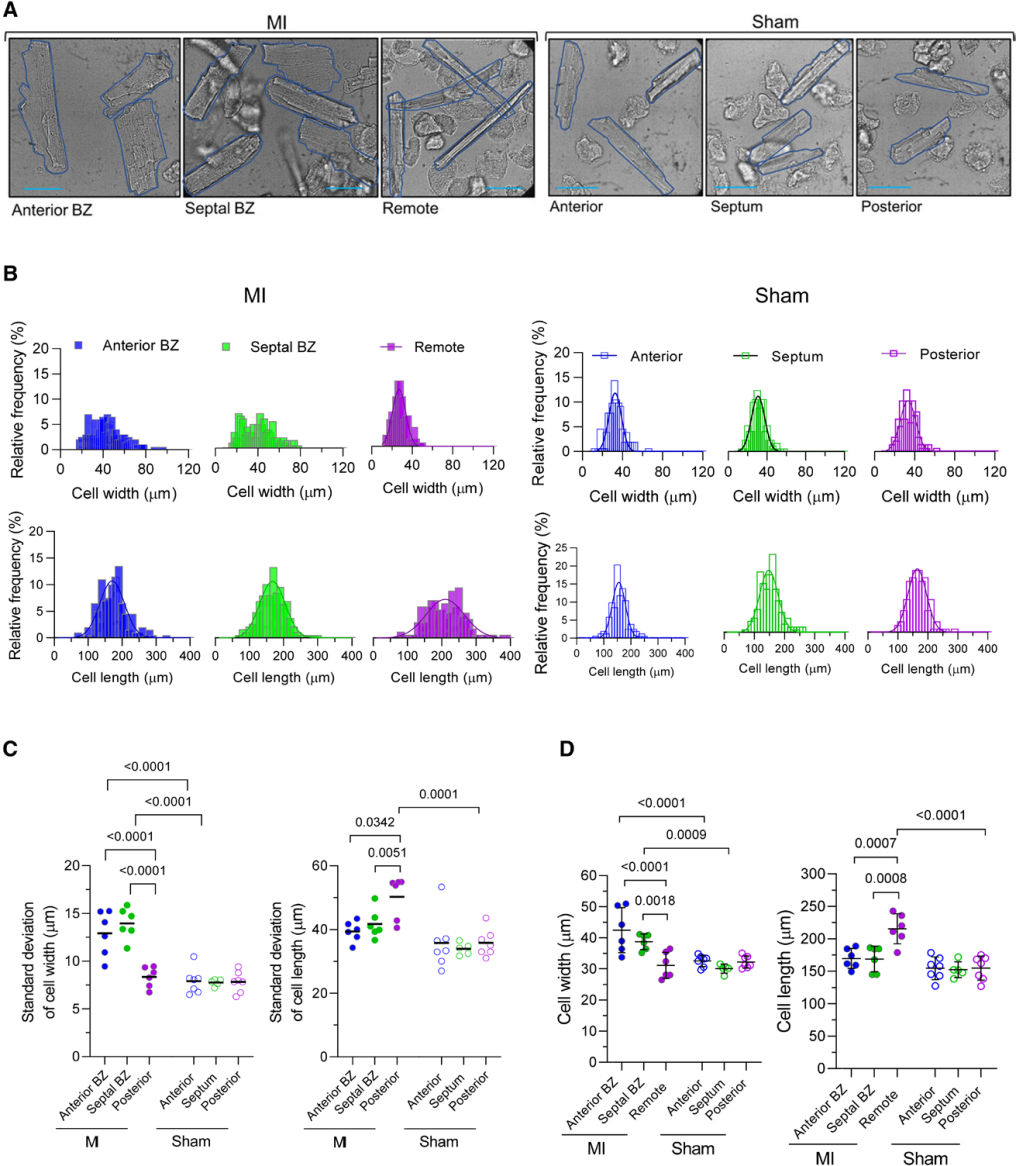
**Heterogeneity of cellular hypertrophic remodeling after myocardial infarction (MI). A**, Examples of isolated cells illustrating regional cell size profiles from an MI pig (**left**) and sham pig (**right**). Scale, 50 µm. **B**, Cell width (**top**) and cell length (**bottom**) distribution by region. Examples of the distribution profile of populations of isolated cells from an MI (**left**) and Sham pig (**right**). **C**, Regional variability of cellular dimensions. **Left**, Variability of cell width and **right**, of cell length, as quantified by the SD per pig from MI (n_pigs_=6, n_cells_=86–216) and Sham (n_pigs_=7, n_cells_=90–330). Mixed-model ANOVA with Bonferroni post test. **D**, Regional differences of cellular dimensions. Mean data of cell width (**right**) and cell length (**left**) per pig from data set in **C**.

Altered repolarization is but one of the phenotypic manifestations of the cellular remodeling that occurs after MI. We hypothesized that variability in repolarization in the BZ would be associated with a similar differential and heterogenous hypertrophic remodeling. To evaluate this premise, we examined cell dimensions and their heterogeneity within and between regions. Representative images of isolated cardiomyocytes from MI and Sham illustrate characteristic morphologies in the different regions (Figure 5A). Figure 5B presents an example of the distribution of cell width and length in matched regions of an MI and Sham pig. The BZ is characterized by a wide distribution of cell width compared with the remote region and with Sham (Figure 5B, top), whereas the cell length appears more variable in the remote region (Figure 5B, bottom). This visual dispersion is confirmed by the greater SD of cell dimensions within each pig (Figure 5C). On average, BZ cells were significantly greater in width but not length, whereas cells in the remote region were greater in length but not width (Figure 5D).

To investigate the relationship between AP variability and cell dimension variability, we also extracted cell length and width from the optical AP recordings presented in Figure 3. A linear regression analysis between matched data for APD and cell size, however, did not detect a correlation between these parameters (R^2^<0.3, *P*<0.05; Figure S5).

### Gene Expression and Cell-Cell Variability of Gene Expression

**Figure 6.**
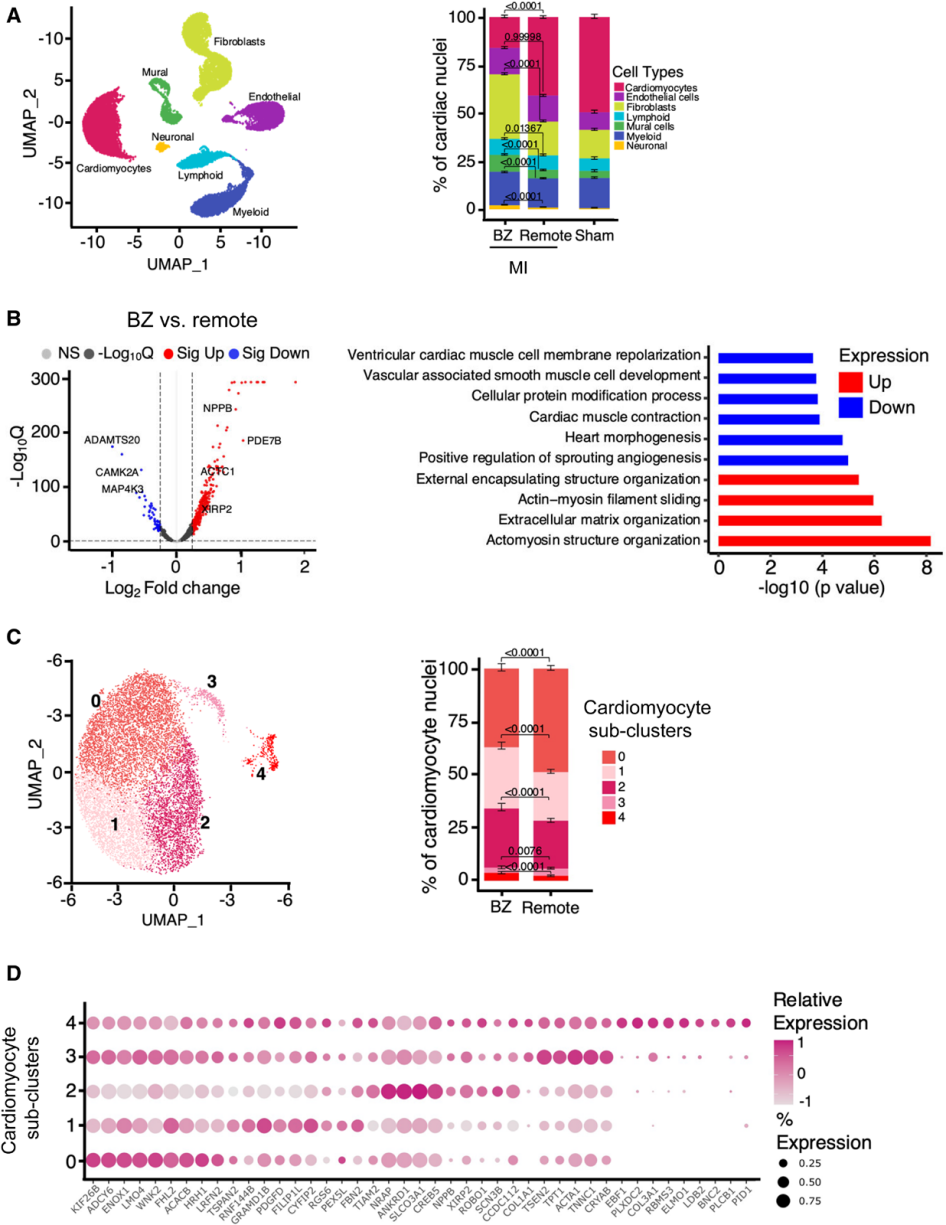
**Border zone (BZ) cardiomyocytes have a unique transcriptomic signature. A**, Major cardiac cell types. **Left**, Uniform manifold approximation and projection (UMAP). **Right**, Fraction of cardiac cell types per region/condition (n_pigs_=3 myocardial infarction [MI], 2 Sham; n_nuclei_=46 028). χ^2^ test with Bonferroni adjustment; error bars represent 95% CI. **B.** Differential gene expression in cardiomyocytes from BZ compared with remote presented as a log to the base 2 fold change (Log2 FC). **Left**, differentially expressed genes (*P*_adj_<0.05; log_2_FC, >0.25 and <−0.25, indicated by dashed lines on the volcano plot) detected with DESeq2. **Right**, Gene ontology and the Kyoto Encyclopedia of Genes and Genomes pathway analysis of the differentially expressed genes. **C**, Cardiomyocyte subclusters and distribution. **Left**, Clustering of cardiomyocyte nuclei from BZ and remote reveals 5 cardiomyocyte subclusters. **Right**, Proportion of cardiomyocyte subclusters in MI BZ and remote (n_pigs_=3, n_nuclei_=10 675). χ^2^ test with Bonferroni adjustment, error bars represent 95% CI. **D.** Gene expression profiles defining the 5 cardiomyocyte subclusters.

The cardiomyocyte AP and hypertrophy data indicate that there are major regional differences in remodeling after MI and also highlight the cellular heterogeneity within the BZ. We considered that the phenotypic heterogeneity within the BZ could reflect heterogeneous influences that via activation of signaling pathways and downstream induction of transcription would lead to cell-cell variability of gene expression. To this end, we performed single-nucleus droplet-based RNA sequencing on total nuclei isolated from the BZ and remote regions from a subset of the MI animals (n_pigs_=3 of both BZ and remote regions), as well as from Sham animals (n_pigs_=2). This analysis yielded 46 028 qualified nuclei. Figure 6A (left) illustrates uniform manifold approximation and projection dimensionality reduction, resulting in clusters representing the different cardiac cell types present in the samples. Nuclei from all samples were represented in all clusters identified, suggesting no batch- or sample-specific effects. As expected, fibroblasts were more numerous in MI than in Sham. This increase in fibroblast proportion was most pronounced in the BZ, which showed significantly greater abundance in comparison with the remote region (Figure 6A, right). Mirroring the increase in fibroblasts, the proportion of cardiomyocytes was significantly decreased in the BZ. Nevertheless, BZ cardiomyocytes were sufficiently numerous (2981 nuclei) to allow a deeper analysis of gene expression profiles.

Cardiomyocyte remodeling in the BZ compared with the remote region was first assessed by differential gene expression analysis (Figure 6B, left). This analysis revealed substantial transcriptional differences (BZ versus remote: up, 425; down, 58; *P*_adj_<0.05; Table S2), characterized by altered expression of genes involved in cardiac hypertrophic remodeling, metabolism, and ion channel function. This difference was further evidenced by the gene ontology and Kyoto Encyclopedia of Genes and Genomes (KEGG) pathways enrichment analysis, showing that pathways of advanced cardiomyocyte function (AP and contraction) were downregulated in BZ cardiomyocytes, whereas pathways involved with myofilament rearrangement and extracellular matrix production were upregulated (Figure 6B, right).

**Figure 7.**
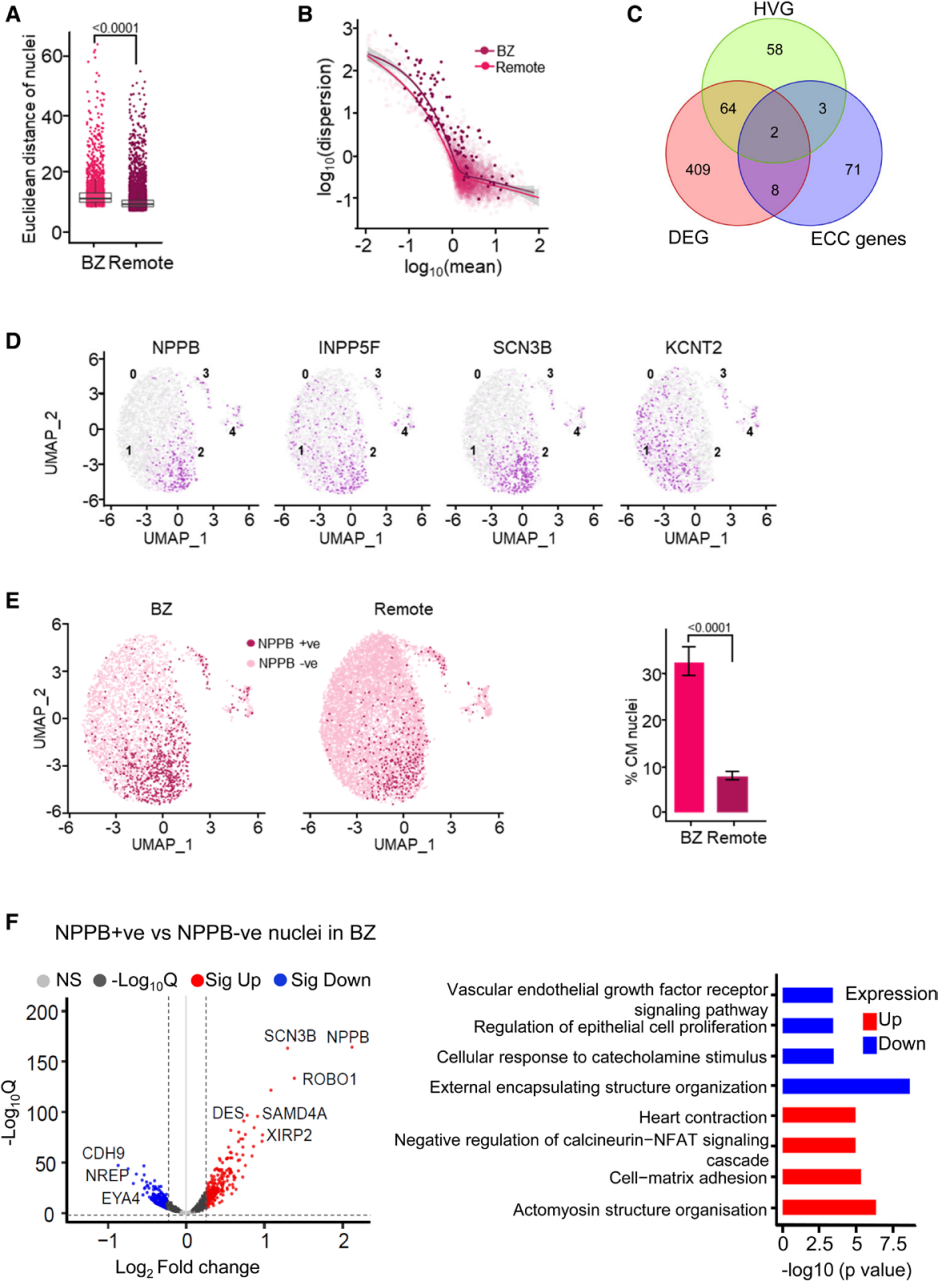
**Cell-cell variability of cardiomyocyte gene expression within the border zone (BZ).** A, Cell-cell variability in BZ cardiomyocytes. Euclidean distances of cardiomyocyte nuclei. n_nuclei_=10 675; n_pigs_=3. Each dot represents a nucleus. Box plots are visualizing the median and interquartile range, and the whiskers are upper/lower quartiles±1.5×interquartile ranges (*P* value refers to a nested *t* test). **B**, Gene expression variability in BZ cardiomyocytes. Dispersion of gene expression in BZ compared with remote cardiomyocyte nuclei. The highly variable genes (HVGs) in BZ compared with remote nuclei are marked with bold dots (expected false discovery rate <0.10, the genes with at least 50% increase in biological overdispersion are defined as HVGs based on a Bayesian inference model). The solid line represents the best fit model of the gene expression variability against expression. (Continued )Figure 7 Continued. The gray bars represent the 95% CI of this model. **C**, Significantly variable genes in the BZ cardiomyocytes. Overlap between the HVGs, differentially expressed genes (DEGs) in the BZ compared with remote presented as a log to the base 2 fold change (Log2 FC) in gene expression on the volcano plot, and excitation-contraction coupling (ECC)–related genes. **D**, Distribution of overexpressed and highly variable genes in BZ. Illustrated are the 4 HVGs, which are also detected as DEGs, across cardiomyocyte subclusters in the BZ. **E**, Regional distribution of Natriuretic Peptide B mRNA expressing nuclei (*NPPB+*). **Left**, Presence of *NPPB+* nuclei shown in uniform manifold approximation and projection (UMAP) of myocardial infarction (MI) BZ and remote cardiomyocytes. **Right**, Fraction of *NPBB+* nuclei in BZ compared with remote (n_nuclei_=10 675, n_pigs_=3 MI). χ^2^ test, error bars represent 95% CI. **F**, Differential gene expression in *NPPB+* vs *NPPB*− BZ cardiomyocytes. **Left**, DEGs in *NPPB*+ vs *NPPB*− nuclei in the BZ (*P*_adj_<0.05; log_2_FC, >0.25 and <−0.25). **Right**, Gene ontology analysis of the DEGs between *NPPB*+ vs *NPPB*− nuclei in the BZ.

To specifically explore cardiomyocyte heterogeneity and cell states, a higher resolution reclustering of this cell type was performed. This analysis identified 5 major clusters representing 5 cardiomyocyte cell states, of which cluster 2 was the most prominent in the BZ, while cluster 0 was significantly lower in BZ than in the remote region (Figure 6C). Notably, cluster 2 showed a high level of mRNA expression of the hypertrophy marker gene *NPPB* (Natriuretic Peptide B), as well as of the stress-related gene *XIRP2* (Xin actin-binding repeat-containing protein 2) and the gene for a mechanosensing protein *ANKRD1* (ankyrin repeat domain 1), which together were recently identified in a cardiomyocyte subcluster defined as stressed cardiomyocytes^22^ (Figure 6D) and previously detected as highly upregulated in the BZ.^23,24^ This cluster was also defined by elevated expression of genes involved in the cardiac cytoskeleton and KEGG terms associated with TGFβ (tumor growth factor-beta) signaling and actomyosin organization (Figure S6A). Clusters 3 and 4 represent the fewest cells in both BZ and remote, although cluster 4 was significantly increased in the BZ at the expense of cluster 3. While cluster 3 was enriched for KEGG terms in transcription/translation, KEGG terms and genes associated with fibrosis were enriched in cluster 4 (Figure S6A). Similar induction of fibrosis-associated genes in cardiomyocytes has been proposed to be associated with proximity to regions of fibrosis.^25^ While these data demonstrate the unique nature of the BZ, this analysis did not directly inform upon the phenotypic heterogeneity within the cardiomyocyte population. We, therefore, probed the gene expression variability between cells within each region and calculated the Euclidean distance between the individual cardiomyocyte nuclei based on the variable genes in the top significant principal components (Figure 7A). Euclidean distances were significantly greater in the BZ than remote indicating greater cell-cell diversity in their transcriptomes. Using a second approach, we assessed variability at the gene level by quantifying the (residual) overdispersion.^26^ One hundred twenty-seven genes showed significantly higher dispersion in the BZ compared with remote, consistent with higher cellular heterogeneity in the BZ (Figure 7B; Table S2). These highly variable genes (HVGs) were associated with KEGG terms including regulation of cardiac muscle hypertrophy, extracellular matrix formation, and MAPK (mitogen-activated protein kinase) signaling (Figure S6B). The HVGs were then compared with the DEG between the BZ and remote and genes associated with excitation-contraction coupling. Figure 7C illustrates that more than half of the HVGs were also differentially expressed between the BZ and remote, including the hypertrophy markers *NPPB* and *INPP5F* (inositol polyphosphate-5-phosphatase F). Further, 2 HVGs, KCNT2 (Potassium Sodium-Activated Channel Subfamily T Member 2) and SCN3B (sodium channel subunit beta 3), which are involved in modulating the action potential, are upregulated in the BZ. Other genes of interest for excitation-contraction coupling are also differentially regulated, including several K^+^ channels such as *KCNJ5* (potassium inwardly rectifying channel subfamily J member 5), which is downregulated (Table S2), but do not show greater variability in expression. Figure 7D maps the 4 differentially regulated HVGs on the BZ cardiomyocyte nuclei and shows that while *KCNT2* is expressed more diffusely, *SCN3B* and *INPP5F* appear to be expressed in cells that also express *NPPB*. These cells are mostly in subcluster 2 (Figure 6C, left). To probe the mechanisms contributing to gene expression patterns observed and examine whether they were driven by common factors, the promoters of the DEG and HVG were interrogated for enrichment of sequence motifs for TF binding. We thus identified binding sites for 173 TFs. Many of these TFs have established roles in cardiac development and disease including GATA-binding factors, nuclear factor of activated T-cells, TEA domain family members, and myeloid ecotropic viral integration site 1. From this group of TFs, we excluded TFs that also potentially regulated DEG and non-DEGs that were not highly variable, yielding 106 TFs that exclusively regulate DEGs that were also HVGs (Figure S7). Among these, only GATA6, for which response elements are found in *SCN3B* and *NPPB*, was increased in expression in the BZ versus remote regions. Together, these data suggest highly specific hypertrophy-related pathways governing alterations observed in the BZ versus remote.

In a next step, we studied the *NPPB*+ cardiomyocytes. Within the BZ, *NPPB*+ nuclei represented a third of all cardiomyocytes, significantly higher than in remote (Figure 7E). When comparing *NPPB*+ versus *NPPB*− cells, we found marked differences in gene expression (Figure 7F, left) BZ *NPPB+* versus *NPPB−*: up, 251; down, 285. Upregulated genes in *NPPB*+ cardiomyocytes include *SCN3B* but also *XIRP2* and *ROBO1* (Roundabout homolog 1). Gene ontology pathways include upregulation of muscle contraction pathways, whereas downregulated pathways include cellular responsiveness to catecholamine stimulus (Figure 7F, right), supported by the KEGG analysis (data not shown).

### Local Drivers for Heterogeneous Remodeling

**Figure 8.**
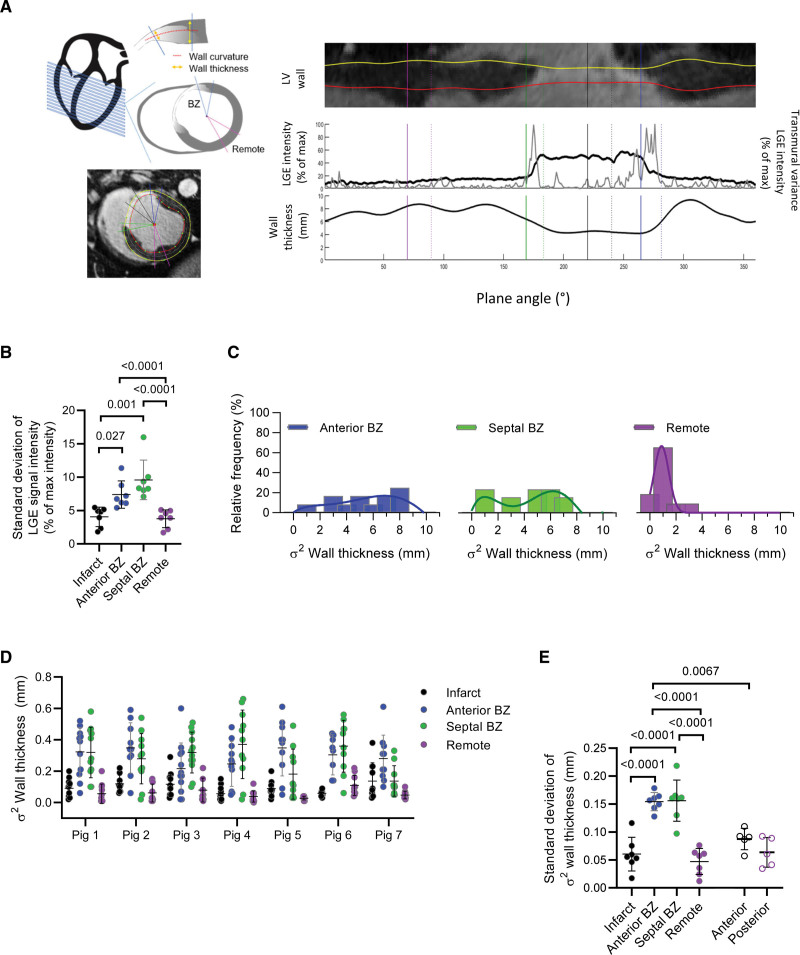
**Heterogeneous regional left ventricular (LV) wall properties after myocardial infarction (MI) in vivo. A**, Measuring local wall stress. **Left**, Illustration of short-axis magnetic resonance imaging (MRI) slice analysis of regional wall properties of thickness and curvature. **Right**, Example of a cardiac MRI (cMRI) midventricular slice demonstrating LV segmentation and corresponding regional wall property analysis based on signal intensity of the late gadolinium enhancement (LGE) signal. **B**, Heterogeneity of scar tissue within the border zone (BZ). Summary data of variability of mean intensity of LGE signal in the cMRI slices from MI (n_pigs_=6, n_slices_=10–14). One-way ANOVA with Bonferroni post test. **C**, Example of distribution of the variability of wall thickness by region in an MI pig. Plot of regional variance (σ^2^) of wall thickness (n_slices_=10–14) illustrating increased heterogeneity in the MI BZ. **D**, Regional variance (σ^2^) of wall thickness in vivo. Data from each slice per MI pig (n_pigs_=6, n_slices_=10–14). **E**, Regional heterogeneity of wall thickness. Summary data of the heterogeneity, quantified by SD from **D**. Mixed-model ANOVA with Bonferroni post test.

Local remodeling after MI is complex involving several factors. We investigated the role of local wall stress. Given a homogenous LV chamber pressure distribution and small variations in wall curvature, wall thickness is the main determinant of wall tension. In the BZ, the number of surviving cardiomyocytes and density of replacement fibrosis is heterogeneous, and hence wall thickness and tension can vary within the BZ. Magnetic resonance imaging data were, therefore, assessed to determine whether local variations in wall thickness were present in our samples (Figure 8A). The BZ characteristically had more heterogeneity in late gadolinium enhancement (LGE) signal intensity, likely reflecting the heterogeneous fibrosis and cardiomyocyte abundance within the BZ (Figure 8B). We defined the heterogeneity by quantifying the variance (σ^2^) of the wall thickness in the radial direction as a metric for local heterogeneity and a stronger assessment than the mean wall thickness. Figure 8C presents the regional distribution of variance of wall thickness in an MI pig with the individual data from each pig presented in Figure 8D (Sham pig data in Figure S8A and S8B). Figure 8E shows that the BZ had a significantly higher variance of wall thickness than remote. Wall curvature did not exhibit a comparable heterogeneity (Figure S8C).

## Discussion

### Heterogeneity of Myocyte Remodeling in the MI BZ and Underlying Mechanisms

In the present data, we found cardiomyocyte heterogeneity at different scales and levels of organization. In comparison to other regions, BZ cardiomyocytes have different but also more heterogeneous morphology, electrical properties, and transcription profiles. The present findings for the BZ as a region are in line with and extend earlier reports of differential remodeling in the BZ compared with the remote region,^12,13,24^ and a recent study^17^ describing heterogeneous repolarization in arrhythmia-sensitive regions at the BZ. Kuppe et al,^22^ using spatial transcriptomics, demonstrated different cell types between BZ and remote in human ischemic cardiomyopathy and local niches of cardiomyocyte types.

Whole-cell patch-clamp recording has been the leading method to study altered cellular repolarization and provides insights into underlying member current alterations. Limitations are, however, the low throughput and concomitantly, the potential for insufficient power to detect small effect sizes, selection bias, and interobserver variability. In addition, the interference with cytosolic content is unavoidable. Optical measurements on the other hand are limited by the parameters that can be assessed but have provided us here with a population view on repolarization for 100s of cells per sample, increasing representability for the heart in vivo and allowing assessment of population heterogeneity.^18^ Single-nucleus RNA sequencing raised the number of cells analyzed another order of magnitude, including 1000s of cardiomyocyte nuclei. While a disadvantage here is the small volume of the biopsy sample, the isolation of nuclei by mechanical disruption from the tissue minimizes cell type isolation biases that may occur when comparing fibrotic and nonfibrotic tissues and thus provides a highly representative picture of the in situ cell gene expression profile.^27^ Across these different scales, analysis of variations within the cardiomyocyte population consistently revealed a higher variability between cardiomyocytes in the BZ than in the remote regions.

Interestingly, across different parameters assessed at the cardiomyocyte level, the findings were not necessarily concordant. At cell phenotype level, cell size did not correlate with APD. At transcript level, *NPPB* expression, previously associated with BZ remodeling after MI,^24^ was a HVG with heterogenous expression in individual BZ cardiomyocytes. It was mostly expressed in a subcluster of cardiomyocytes identified as cluster 2, where also *SCN3B* expression was higher. *SCN3B* encodes a subunit of the cardiac Na^+^ channel that modulates the late Na^+^ current and thus APD.^28^ Variable expression thus could contribute to APD heterogeneity. For other ion channels, the distribution was different. *KCNT2* also displayed highly variable expression but with different clustering than *SCN3B*, whereas *KCNJ5*—a gene encoding for a GPCR (G-protein coupled receptor)-modulated K^+^ channel—was more homogeneously downregulated. The latter channel may be underlying the reduced inward rectifying current and lability of the resting membrane potential during adrenergic challenge that we observed previously.^15^ Altogether, combinations of altered gene expression could lead to the phenotypic heterogeneity that contributes to increased arrhythmia susceptibility. These complex changes in gene expression can result from variable activation of signaling pathways and the TFs they converge upon. Of note, GATA6, which was upregulated in the BZ, is necessary and sufficient for induction of pathological hypertrophic remodeling and acts in a dosage-dependent manner.^29^ This makes GATA6 a likely candidate to contribute to the heterogeneity in gene expression. Of further interest, EBF1 (Early B-cell Factor-1), driving the expression of several of the DEGs, is itself an HVG.

### Drivers of Heterogeneous Cell Remodeling Within the BZ

The BZ is a highly complex environment where many factors can initiate signaling for cardiomyocyte remodeling. During coronary occlusion, the transitional zone between the core ischemic and the fully perfused area is in a state of relative ischemia and is associated with cell death and remodeling, leading to the formation of the future BZ.^30^ During reperfusion, further cell death and remodeling occurs involving activation of survival pathways and inflammation, which initiates a cascade of events that lead to infarct healing.^31^ However, whether inflammation and signaling from inflammatory cells is variable within the BZ and thereby contributes to cardiomyocyte heterogeneity is not known, although recent data highlight the rich cellular environment.^22,25^ In our data as well, myeloid and lymphoid cells are highly enriched in the BZ. Of note and in line with inflammation also being heterogeneous, a subpopulation of cardiomyocytes (subcluster 2) showed enrichment of KEGG pathways associated with TGFβ signaling. Similarly, recovery of neurons and heterogeneous nerve ending growth leading to alteration in cardiomyocyte innervation can contribute to the variability in individual cardiomyocyte phenotype.^32,33^ The presence of a cell population with neuronal characteristics in the BZ, with a density double that in the remote zone (Figure 6A), supports this concept. Isolation of these cells is a challenging project for future study.

We additionally identified mechanical load and stretch, as a driver of cardiomyocyte heterogeneity as we found significant variation in wall stress along the BZ. The differences in local wall thickness that are responsible for this variable wall stress can be the consequence of variability in the relative perfusion, cell death, inflammation, and replacement fibrosis. There is a well-established link between stretch-induced hypertrophy and local production of angiotensin II, by cardiomyocytes and fibroblasts.^34^ The observation of high levels of mRNA expression for XIRP2 (also known as Xinβ and paralogue of XIRP1/Xinα), a protein involved in mechanosensing, cell-cell interaction, and component of the intercalated disc, supports the hypothesis that local differences in wall stress contribute to local differences in hypertrophic remodeling. Further, XIRP2 expression is regulated by MEF2A (myocyte enhancer factor 2) in angiotensin II–induced cardiomyocyte hypertrophy.^22,35^ Notably, and further linking it to remodeling, *XIRP2* functionally interacts with Hippo-Yes Associated Protein (YAP) signaling during cardiac development and disease, including in humans.^36^ Of interest, the areas with the highest local wall stress coincide with areas of fibrosis. Recently, a stressed cardiomyocyte gene expression pattern was localized to areas of fibrosis in ischemic cardiomyopathy,^22^ suggesting convergence of mechanical stress and cell-cell signaling in a microenvironment as source of heterogeneity.

### Contribution of Myocyte Heterogeneity to In Vivo Repolarization Heterogeneity and Relevance for Arrhythmia

Spatial and temporal heterogeneity of repolarization in the heart facilitates arrhythmias, predominantly as a substrate for reentry. Our finding that heterogeneity of repolarization in the BZ was related to arrhythmogenicity is in line with clinical studies, where the BZ has areas that were vulnerable to reentry during programmed electrical stimulation to sustain arrhythmias.^3,37^ These vulnerable areas have mostly been seen as regions of irregular fibrosis, identified in magnetic resonance imaging and supported by computational studies.^2,38^ Our data suggest that cell-cell variability contributes to the vulnerability. Such a link is supported by the correlation between cell-cell heterogeneity and ARI heterogeneity (Figure 4C) and the association between ARI heterogeneity and vulnerability to arrhythmias (Figure 2E). While the heterogeneity of repolarization seen in populations of isolated cardiomyocytes ex vivo is expected to be dampened in vivo by cell-cell coupling, the ARI data indicate that it is preserved at the tissue level. In the BZ, reduced and lateralized connexin and gap junction expression and presence of fibrosis may result in less efficient coupling and thereby enhance the contribution of cell-cell heterogeneity to the local ARI heterogeneity.^39,40^ On average, the ARI durations in vivo were shorter than the cellular APDs despite correction of the ARI for heart rate. It is likely that while cellular AP recordings reflect the cell’s native ion channel composition, in vivo circulating catecholamines, electrolytes, drugs, mechanical loading, interactions with the matrix, and autonomic innervation modulate the resultant APs.^41^

Arrhythmia susceptibility is multifactorial. As the data suggest that high wall stress in thin fibrotic areas can underlie cardiomyocyte variability, at least 2 mechanisms, heterogeneous cardiomyocyte remodeling and local fibrosis, likely conspire to create in vivo vulnerable regions.

### Limitations

The study aimed to assess heterogeneity across different scales, but technical limitations caution for a full correlation of data. The electroanatomical mapping performed in vivo utilized an endocardial approach that does not provide information on transmural and epicardial heterogeneities, while the cellular isolation reports on the bulk mid-myocardial layer. Moreover, we did not perform atrial pacing maneuvers or investigate the effects of heart rate on regional ARI in vivo, which should be a consideration when interpreting the corroborative in vivo–in vitro data.

Our gene expression studies yield insights into cellular remodeling that could explain the manifest AP heterogeneity; however, it should be considered that beyond gene expression, mRNA translation and posttranslation modifications of proteins will occur that through alterations in protein abundance and activity, respectively, also contribute to the eventual AP heterogeneity. Future studies allowing large population studies of ionic currents under patch-clamp^42^ and cell-cell analysis of protein abundance/modifications are needed to address this question.

### Conclusions

Electrical and hypertrophic remodeling of cardiomyocytes in the BZ is heterogeneous, with underlying cell-cell variability of gene expression. Differences in local wall stress and in microenvironment of innervation and inflammation likely drive this heterogeneous remodeling. The cell-cell heterogeneity contributes to heterogeneous repolarization in vivo and arrhythmia vulnerability within the BZ.

## Article Information

### Acknowledgments

The authors thank Patricia Holemans and Roxane Menten for expert technical assistance with the experimental data acquisition and animal care and Annemie Biesemans for assistance with cell isolation. With great sadness, the authors inform of the death on October 3rd, 2022 of the first author and driver of this study, Matthew Amoni.

### Sources of Funding

This work was supported by the FWO, the Fund for Scientific Research-Flanders (FWO grant number G097021N to Drs Sipido, Willems, Roderick, and Claus; G0C6419N to Dr Roderick; Dr Willems is supported as Senior Clinical Investigator and Dr Amoni as PhD fellow) and by the Special Research Fund, BOF, of KU Leuven (to Drs Willems, Claus, grant number C14/18/079, and Roderick, grant number C14/17/088).

### Disclosures

Dr Willems reports research funding and speakers and consultancy fees from Abbott. The other authors report no conflicts.

### Supplemental Material

Supplemental Methods

Tables S1–S2

Figures S1–S8

References ^43–57^

## Supplementary Material
