# Supplementary material for: Heterogeneity of Repolarization and Cell-Cell Variability of Cardiomyocyte Remodeling Within the Myocardial Infarction Border Zone Contribute to Arrhythmia Susceptibility
Source: Circ Arrhythm Electrophysiol. 2023 May 2;16(5):e011677. doi: 10.1161/CIRCEP.122.011677 (PMC10187631; doi:10.1161/CIRCEP.122.011677)
Supplement: Supplementary file 1 [file hae-16-e011677-s001.docx]

**Heterogeneity of Repolarization and Cell-Cell Variability of Cardiomyocyte Remodeling Within the Myocardial Infarction Border Zone Contribute to Arrhythmia Susceptibility**

# Supplemental Methods

## Animal handling

The experimental protocols of this study were approved by the KU Leuven Animal Ethical Committee (Ethische Commissie Dierproeven, KU Leuven), with permit number ECD137/2018. Animal husbandry and treatment was according to the Guide for the Care and Use of Laboratory Animals (National Institute of Health, USA), and the European Directive 2010/63/EU.

Thirty-four domestic pigs (*Sus scrofula domesticus* Strain: TN70, Topigs Norsvin) of both sexes (females and castrated males) were used in this study. We aimed for groups of 6 animals per analysis data set for *in vivo* and *ex vivo* measurements, based on previous experience to detect differences between border zone and remote regions, but without formal sample calculation given the exploratory nature of the study. Swine were of weight 34±4 kg on entry into the study and were randomized between MI (N = 22) and Sham (N = 11) in consecutive order during the experimental planning. Two animals were excluded from the final dataset due to sudden cardiac death soon after the induction procedure. Thirteen animals used in this study were also used to gather data presented in a recent study^15.^

Animals were housed in single pens with a standard 14 h light, 10 h dark cycle, temperature of 22 ± 2°C and humidity of 45-70%. Animals had free access to a standard chow (Optivo Pro 9041, AVEVE, Belgium) and water. Exception was made in the week of experimentation; animals were fed twice a day (between 7 and 8 am and between 12 and 2 pm, with welfare monitoring), and fasted the day before *in vivo* procedures.

All invasive procedures and *in vivo* experiments were performed under general anesthesia. The anesthetic regime consisted of an initial sedation with tiletamine/zolazepam 8 mg/kg and xylazine 2.5 mg/kg intra muscular (IM). The animal was then transported to the laboratory where intravenous access was obtained. Then, anesthesia was induced with propofol 3 mg/kg, and maintained with a combination of propofol 10 mg/kg/h and remifentanil 0.2-0.4 µg/kg/h intravenously (IV) introduced. Animals were intubated endotracheally with 6-8 Fr standard endotracheal tube and ventilated with a 50% oxygen-air mixture at 6-10 mL/kg tidal volume. During all invasive procedures, antibiotics (cefazolin 22-50 mg/kg, IV) and anticoagulation (acetylsalicylic acid, 500 mg and periodic heparin 5000-10000 IU, IV) were routinely administered. Post-operatively, animals received broad-spectrum antibiotics (amoxicillin 15 mg/kg or enrofloxacilin 4 mg/kg) with analgesia (buprenorphine HCl 0.02-0.03 mg/kg, IM and fentanyl transdermal patch 12 µg/h for 72 h).

The full *in vivo* experimental protocol is illustrated in Figure S1A.

## Myocardial infarction induction

A clinically relevant ischemia-reperfusion model of MI was used as previously described. Briefly, all animals were pre-medicated for three days with amiodarone 300 mg, as well as one day of aspirin 300 mg and clopidogrel 300 mg. Animals were then randomized to either MI induction or Sham operation on day 0. Under anesthesia, the left carotid artery was directly cannulated by surgical cut-down with a 5/6 Fr vascular sheath. Baseline left ventricle (LV) and aorta pressures were recorded using a Millar pressure catheter and PowerLab system (ADInstruments, AU). Then, under fluoroscopic guidance a 5/6 Fr Judkins-left or Amplatz-left coronary guiding catheter was positioned in the left main coronary ostium. An intracoronary injection of nitroglycerine 250-300 mg was administered intracoronary to prevent vasospasm. 5 mg/kg amiodarone IV was administered as a bolus to reduce arrhythmias. A standard 0.014-inch coronary wire was stabilized in the left anterior descending coronary artery (LAD) and a 2.5-3.0 mm angioplasty balloon, was positioned over-the-wire immediately after the first diagonal branch (D1) and completely inflated to ~12 bar to induce ischemia of the anterior-septal territory. Complete occlusion of the distal 3/4 of the LAD was confirmed by angiography and was also evident from ST-segment elevation. Ischemia, was maintained for 120 min after which, the balloon was deflated and the myocardium reperfused. The animal was monitored for at least 60 min and the carotid artery repaired. Sham animals, underwent the same procedure, without occlusion, and were included as controls.

During ischemia, sustained ventricular tachycardia or fibrillation, occurred in approximately 17/22 (77%) of animals and was managed by prompt external defibrillation with 150-200 J to restore sinus rhythm. Hypotension (BP < 60/40 mmHg) was treated with noradrenaline 2-5 µg/min or adrenaline 1-10 µg/min infusion and weaned during reperfusion. All animals (MI and Sham) received amiodarone for 3 days, aspirin 100 mg and clopidogrel 75 mg was given daily for 1 month.

No animals died during the MI induction. The early mortality (<5 days post-MI) was 9% (2 animals), due to SCD after MI. After ~5 days of observation, animals were transferred to the KU Leuven University farm and returned ~ day 28 post-MI.

## Cardiac magnetic resonance imaging

One month after MI or sham procedure, animals were brought under general anesthesia and cardiac magnetic resonance imaging (cMRI) was performed using a 3T whole-body scanner (PRISMA, Siemens, Erlangen, Germany) as previously described^15^. Animals were placed supine in the scanner, electrocardiogram (ECG) leads were placed around the heart and a two phased-array body coils were wrapped around the thorax to increase signal to noise ratio. Acquisition was performed with ECG-gating during periods of apnea, achieved by suspending mechanical ventilation. Standard clinical sequences were adapted to acquire comprehensive views of the heart as well as cine images in short axis (SAX) views for analysis of function. Corresponding images of late gadolinium enhancement (LGE) were acquired 12 min following an intravenous bolus of Gadolinium (gadoterate meglumine, Dotarem, 0.2 mmol/kg).

The cMRI data was analyzed and images segmented with custom software (RightVol; KU Leuven) on an offline workstation to assess heart structure, dimensions and function as well as left ventricle (LV) infarct size. Data are summarized in Supplemental table 1.

To assess local variability of LV wall properties in different LV regions and compare the BZ to remote, we analyzed the wall thickness, curvature and LGE of each slice. On each SAX slice covering the scar, the BZ was defined as the region with signal intensity 20-70% of the maximum intensity (infarct core), and the remote as the posterior wall opposite to the BZ (Figure 8A). The myocardial curvature and variance (σ^2^) of wall thickness of each region were calculated as previously^43^.

Two to four days after the cMRI, an *in vivo* electrophysiology (EP) study was performed.

## Cardiac electrophysiology study

The *in vivo* electrophysiological study was performed under general anesthesia as before, with the exception that sedation was achieved with tiletamine/zolazepam 8 – 12 mg/kg only, omitting xylazine to avoid its adrenergic effects. Vascular access for cardiac catheterization, was obtained by cannulating two arteries (carotid or femoral) and two veins (jugular or femoral). Carotid and jugular veins were exposed by surgical cut-down and femoral vessels were punctured percutaneously under ultrasound guidance. An 8 or 9 Fr vascular sheath was used for arterial access and 7 Fr sheaths for venous access. A single bolus of aspirin (500 mg), and periodic boluses of heparin (5000-10000 IU) were administered for anticoagulation.

Follow-up, LV and aortic pressures were recorded as described above. Then, electroanatomical mapping of the left ventricle (LV) endocardium was performed using the EnSite Precision 2.0 mapping system (Abbott Medical, New Jersey, USA) paired with a BARD Electrophysiology Labsystem Pro EP (Boston Scientific, USA) as described previously^1^. Standard electrophysiology catheters (Biosense Webster, Diegem, Belgium) were positioned in the coronary sinus and the right ventricle (RV) apex for orientation and diagnostic signals. The 9 Fr 64-electrode multi-electrode array (MEA) catheter for non-contact mapping was positioned centrally in the LV chamber under bi-plane fluoroscopic guidance, with the pigtail in a stable position in the LV apex.

A bi-plane left ventriculogram (left anterior oblique 50° and right anterior oblique 40°) was made under fluoroscopy with 60-80 ml of a 50-80% saline-iodine mixture, to estimate chamber dimensions and guide mapping. An electroanatomical map of the LV was created by roving the LV using a decapolar Lasso and quadripolar linear mapping catheter paired with the MEA. Point-by-point contact mapping was performed with attention to acquiring bipolar voltages annotated on the map to create an electroanatomical map of the LV. This was used to define the conventional BZ using peak-to-peak voltages of 0.5-1.5 mV (Figure S1A).

When the mapping procedure was completed, the mapping catheter was withdrawn from the LV. After a stabilization period of approximately 20 min, the sinus electrical activity of the LV was recorded by non-contact mapping for 5 min. For non-contact mapping, electrograms in 2048 virtual electrode positions equally distributed around the LV anatomical map were continuously calculated in real-time by the Ensite mapping system through inversion of the 64 MEA electrode signals. Virtual electrograms were recorded at a sampling rate of 1.2 kHz and filtered with a bandwidth of 0.1-300 Hz.

After the EP study, surgical access was repaired and hemostasis achieved, after which the animal recovered with appropriate analgesia (buprenorphine HCl 0.02-0.03 mg/kg, IM) and antibiotics (amoxicillin 15 mg/kg or enrofloxacilin 4 mg/kg, IM).. Procedure-related mortality after EP study was ~16% (4 MI and 2 Sham).

### In vivo mapping data analysis by E-field

All electroanatomical and electrophysiological data were exported (EnSite Velocity System v.5.2 software) to an offline workstation and analyzed using a custom-made Matlab software (RightHemo-MEA, KU Leuven). Activation-repolarization intervals were calculated for each of the 2048 virtual electrogram positions on the endocardial surface with a recently developed and validated method for analysis of densely distributed electrograms.^19^

The local ARI durations for each annotated electrogram were visualized as a colormap on the 3D endocardial surface as well as on a polar map (Figure S2B). To quantify local ARI heterogeneity, a heterogeneity index was determined for each electrogram as the standard deviation of ARIs within a circle with 1 cm radius surrounding each electrograms (Figure 1B middle). This index was also visualized as a colormap on the 3D endocardial surface and as a polar map to highlight regions with increased ARI heterogeneity (Figure S2C).

### VT inducibility index

The inducibility of VT/VF as a surrogate of arrhythmia vulnerability was assessed using a scale constructed from the stepwise progression of the programmed electrical stimulation protocol where the first step (S1 = 600ms and 1 extrasystole, S2 = 400 ms) was 100% and the last most aggressive step (S1 = 350 ms and 3 extrasystoles, S2 = 200 ms, S3 = 180 ms, S4 = 150 ms) marked non-inducibility as 0%, adapted from^44^.

## Sacrifice and cell isolation

After 1-3 days for recovery, animals were sacrificed for tissue sampling and cell isolation as previously^15^. The full experimental protocol for *ex vivo* studies is illustrated in Figure S1B.

For sacrifice, the animals were sedated, and anesthesia was induced with pentobarbital (20 mg/kg, IV) with artificial ventilation. A midline-sternotomy, was performed and the heart exposed by opening the pericardium and mobilized. Full anticoagulation with heparin (20 000 IU) was given and an overdose of anesthesia was given (50 mg/kg pentobarbital, IV). The heart was quickly harvested by dividing the great vessels and placed in cold oxygenated Tyrode’s solution on ice and transported to the lab.

All hearts were prepared by rinsing with cold oxygenated Tyrode’s solution. The heart was then dissected to isolate the LV. From MI hearts, LV wedges of the anterior BZ, the septal BZ and the non-infarcted remote region (Figure S1B) were dissected and their perfusing arterial branches cannulated as follows: anterior BZ via the proximal LAD above the 1^st^ diagonal branch and the septal BZ the entire LAD above the 1^st^ conus branch with ligation of leaking branches, finally the remote region was cannulated via the left circumflex or obtuse marginal branch. In Sham hearts, the matching regions from the anterior, septal and posterior LV wall were chosen. A small piece (0.5-1 g) of the anterior BZ, septal BZ and remote territories used for cell isolation were collected and snap frozen in liquid nitrogen and stored at -80 °C for batch processing.

The wedges were perfused by a constant flow Langendorff system (3-4 ml/min) at 37 °C. The tissue was briefly perfused with normal Tyrode’s (in mmol/L: NaCl 137, KCl 5.4, MgCl_2_ 0.5, CaCl_2_ 1.8, Na-HEPES 11.8, and glucose 10; pH 7.4) to washout residual blood. Then the tissue was perfused with Ca^2+^-free Tyrode’s, followed by enzyme-containing Ca^2+^-free Tyrode’s solution (collagenase-A, Roche and protease-XIV, Sigma Aldrich). After digestion, low Ca^2+^ Tyrode’s (in mmol/L: NaCl 130, KCl 5.4, KH_2_PO_4_ 1.2, MgSO_4_ 1.2, CaCl_2_ 0.18, Na-HEPES 6, glucose 20, pH 7.2) was perfused to wash out the enzyme.

From the BZ wedge preparations, a strip was dissected that contained the transition from scar to healthy myocardium (Figure S1B). The digested tissue samples from the BZ and remote region were minced and filtered to liberate single cardiomyocytes into the solution. The isolated cardiomyocytes were then resuspended in normal Tyrode’s.

## Cellular electrophysiological recordings

Cellular action potentials were recorded from the isolated cells by patch-clamp techniques as before^13^. Cells were placed on the stage of an inverted microscope with a setup as previously described^4^. Cells were constantly superfused with normal Tyrode’s solution at 37 °C. Patch pipettes were fashioned from borosilicate glass (GB 200-8P, Science Products) and pulled to a resistance of 2-3 MΩ when filled with internal solution (in mmol/L): K-aspartate 120, NaCl 10, KCl 20, K-HEPES 10, MgATP 5, and K_5_Fluo-4 0.05; pH 7.2. Whole-cell patch clamp mode was employed and cells studied using the current clamp configuration (Axon 200B amplifier, Axon Instruments). Cells were stimulated with a suprathreshold depolarizing pulse of 2-4 nA, until steady state, after which at least 30 APs were recorded.

Patch-clamp data were exported in .abf format and files were analyzed using pClamp software v11 (Molecular Devices, USA). Cell capacitance, resting membrane potential and AP duration (APD) at 90% repolarization (APD90) were analyzed.

For population studies, optical recordings of APs were performed using a voltage-sensitive dye. An aliquot of the freshly isolated cells was incubated at 37 ˚C for 30 min with 5 µM Di-8-Anepps (Thermo Fisher Scientific, Belgium), then washed and placed in a recording chamber of an inverted microscope (Nikon Ti-E, Germany), equipped with a 40x oil immersion objective lens, N.A. = 1.3 and refractive index = 1.5. The cells were superfused with Tyrode’s solution at 37 ˚C and field-stimulated with suprathreshold 2-4 ms pulses at 1 Hz, generated by 2 platinum electrodes placed in the bath. A CoolLED pE340^fura^ illumination system was used for excitation centered at 488 nm, with emission recorded at >535 nm. Fluorescence emission was captured using a sCMOS camera at 200 Hz with an exposure time of 5 ms and 4×4 binning, with resultant voxels of 0.65 μm/pixel (Orca flash-c11440, Hamamatsu). Illumination and image acquisition were controlled with Nikon NIS-Elements (v5) software.

Image files were exported in .ND format. Recordings were analyzed in ImageJ software (NIH, USA) using a semi-automated custom-written macro. Briefly after a pre-processing step, the cells were semi-automatically segmented and identified; then fluorescence changes associated with the AP within the region of interest were converted to an AP waveform. APs were then automatically analyzed in MatLab. The last 10 APs were averaged and the APD at 25% (APD25), 50% (APD50) and 90% repolarization (APD90) were collected.

## Single-nucleus RNA sequencing and gene expression analysis

Isolation of nuclei from frozen tissue was selected to avoid many artefacts associated with transcriptome analysis in enzymatically digested cells. When isolating nuclei from frozen tissues, biases in cell type isolation and in isolation of cells from more difficult to digest fibrotic tissue is avoided. Many studies now use this approach for the aforementioned reasons, and we have established the method in our lab previously.^45^ As nuclei are isolated from frozen tissue, all samples can be processed in parallel thereby avoiding batch effects. The isolation of nuclei from snap frozen tissue (in the presence of actinomycin D to inhibit transcription) ensures that a transcriptome that reflects the cell state at time of tissue freezing is captured. This contrasts with transcriptomes from enzymatically digested tissues where transcription continues during the 1 h or so of tissue digestion and induction of stress related immediate early genes is reported. Nuclear RNA-Seq does however capture more non processed RNAs and more intronic sequences. While this is ideal for determining the genes that are being transcribed at the time of harvest, effects on mRNA abundance due to differential rates of mRNA degradation via miRNAs and RNA binding proteins are missed.

Tissue samples collected under RNase free conditions from the same regions as used for cell isolation were rapidly frozen in liquid nitrogen and stored at -80 °C.

### Isolation of cardiac nuclei

Debris-free suspensions of intact nuclei were generated by tissue homogenisation followed by sucrose gradient centrifugation and flow sorting with all steps performed at ≤ 4 °C with modifications from Thienpont *et al.,*^45^ and Preissl *et al.,*.^46^ Prior to processing for nuclear isolation, sample quality and preservation were assessed by Bioanalyzer analysis of RNA isolated from the samples used. Only samples with an RNA Integrity (RIN; Agilent Bioanalyzer) of > 7.6 were used. Approximately 80 mg of snap-frozen cardiac tissue (each sample in duplicate) was cut on ice with a scalpel into 4-5 smaller pieces after which they were placed into gentleMACS M-tubes (Miltenyi Biotec, 130093236) containing 5 mL lysis buffer and thawed on ice (5 mM CaCl_2_, 3 mM MgAc, 2 mM EDTA, 0.5 mM EGTA, 10 mM Tris-HCL pH 8, supplemented prior to use with 1 mM DDT (Sigma-Aldrich, 646563), 1 µg/mL actinomycin D (Sigma-Aldrich, A1410), 0.05% protease inhibitor cocktail (Sigma-Aldrich, P8340), and 0.04 U/μL RNA inhibitors-RNase OUT (Thermo fisher Scientific, 10777019)). Tissue was then homogenized using a gentleMACS dissociator (Miltenyi Biotec) on program “protein_01” for 1 min. After homogenization, 5 mL lysis buffer supplemented with 0.1 % NP-40 Surfact-Amps™ (Thermo fisher Scientific, 28324) was added to the cell suspension, which was then filtered through a 30 μm filter to remove non-disrupted tissue and cells (Miltenyi Biotec, 130041407). To recover retained nuclei, the filter was washed with an additional 2 mL lysis buffer containing 0.1 % NP-40 Surfact-Amps™. Nuclei were then harvested by centrifugation (1000 x g, 5 min), after which the pellet was resuspended in and overlayed on sucrose buffer (1 M sucrose, 3 mM MgAc, 10 mM Tris-HCl, pH 8) containing 1 mM DDT, 1 µg/mL actinomycin D, 0.05% protease inhibitor cocktail, 0.04 U/μL RNA inhibitors-RNase OUT, and 2 µg/mL WGA (Sigma-Aldrich, L9640), then pelleted by centrifugation (1000 x g, 5 min). The cardiac nuclei pellet was washed twice (650 x g/ 500 x g, 6 min) in PBS including 0.04 U/μL RNA inhibitors-RNase OUT, 2 µg/mL WGA, and 750 µg/mL UltraPure™ BSA (Thermo fisher Scientific, AM2616), after which nuclei were resuspended in staining buffer (wash buffer containing 0.02 mg/mL DAPI) for 10 min. After centrifugation (500 x g, 6 min), the pellet was resuspended in 300 μl PBS, pooled with the duplicate pellet of each sample, and passed through a 35 µm cell strainer. Cardiac nuclei were sorted into PBS containing 0.04 U/μL RNA inhibitors-RNase OUT and 750 µg/mL UltraPure™ BSA using a BD Influx flow cytometer. Intact debris-free cardiac nuclei were harvested by setting gates for DAPI+ve and particle size. Finally, after counting using a LUNA-FL™ Counter (Logos Biosystems), cardiac nuclei were pelleted by centrifugation at 500 x g for 6 min, and then resuspended into sorting buffer.

### Sequencing and analysis of snRNA-seq data

Cardiac nuclear suspensions (~6000 nuclei per animal, based on counting) were loaded on a Chromium Single Cell Instrument (10x Genomics) to generate single-nucleus GEMs. Single-nucleus RNAseq libraries were prepared using Chromium Single-cell 3’ Library Version 3 Chemistry, Gel beads, Chip, and Multiplex kit (10x Genomics) following the manufacturer’s protocol. Libraries were sequenced on Illumina Novaseq 6000 at a depth of 30,000 reads per nucleus.

### Single nucleus transcriptomics analysis

Transcriptome datasets from sequenced reads per lane (each lane contains only one animal sample) of sequencing were provided by CellRanger V5 through aligning the reads to both introns and exons from premature unprocessed nuclear RNAs. Ambient RNAs and low complexity libraries in the raw expression matrices were removed with CellBender^47^ and nuclear doublets detected and removed by scDblFinder^48^. Singlet nuclei from different datasets were then merged and further processed using Seurat V4^49^ in R V4.1.2 (R Core Team (2021)). Nuclei were further filtered according to following QC criteria: These extra QC thresholds are adjusted based on the log10 nGenesPerUMI (> 0.8) as a library complexity index, nUMI (<60000), nGenes (>350) based on the outliers of log10X visualized by boxplot, and the percentage of reads originating from mitochondrial coded RNAs (<5%) and rRNAs (<5%). After library normalization, gene expression scaling, and identification of variable genes, the nearest neighbour graph was constructed based on the Euclidean distance of the nuclei in each top significant principal component (PCs). Next, the main cell types are clustered using the Louvain algorithm at a low resolution (0.1). To visualize clusters, the UMAP (Uniform Manifold Approximation and Projection) non-linear dimensional reduction approach was used to group similar nuclei in a low-dimensional space. Thereafter, the clusters were annotated based on their expression of established cell-type-specific marker gene expression.^20^ Cardiomyocyte nuclei with > 700 genes detected were then extracted from the whole merged datasets and integrated using Harmony^50^ and re-clustered at a higher resolution (0.25). The proportion of each cardiomyocyte subtype detected was then quantified per heart region and the ratio of cardiomyocyte cluster 0 to other cardiomyocyte subtypes in each region statistically compared (Chi Square test).

The genes defining each cardiomyocyte subtype were identified amongst those genes expressed in at least 10% of nuclei in either of the two groups of cardiomyocyte subtypes and visualized by dittoDotPlot.^52^ The differentially expressed genes in all BZ vs Remote cardiomyocyte nuclei were then determined by DESeq analysis and further analysed by enrichR^53^ to identify affected biological pathways. The differentially expressed genes were visualized by an Enhanced Volcano plot.^54^

We analysed 2981 cardiomyocyte nuclei from the BZ, and 7694 cardiomyocyte nuclei from the remote regions with a mean of 3255 genes per cell for the BZ and 3055 genes per cell for the remote region.

To analyse cellular heterogeneity in each heart region, two approaches were taken. First, to determine heterogeneity at a cellular level, the mean of the Euclidean distance of each cell relative to other cells in each region (per animal) was calculated based on the expression of variable genes in the top 13 significant principal components, which defined the cell subclustering. The data were visualized using ggplot2/ tidyverse^55^ and statistically compared using a hierarchical t-test.

To identify variation at the level of genes, the BASiCS^57,26^ algorithm was used to determine the significantly (residual) over-dispersed genes after removing very lowly expressed genes (MinCellsWithExpression=2 and MinTotalCountsPerCell/Gene=2). The significant expressed/dispersed genes were identified with an expected false discovery rate (EFDR) < 0.10.

The Venn R package was used to illustrate the overlapped genes in each list of interest. For visualizing the distributed nuclei expressing the candidate genes, the FeaturePlot() function in Seurat package was used. Of note, each individual plot is scaled to the maximum expression value of the gene across the conditions/regions.

## Analysis and statistics

Data acquisition was performed by multiple investigators and investigators were blinded when doing analysis off-line by coding or anonymizing data files.

All data are presented as means ± SD or median (IQR) and mostly presented by animal as indicated in the figure legends. Distribution of data was tested by Shapiro-Wilk to determine appropriate statistical tests.

Data were compared with unpaired t-test or a mixed-effects model 1-way or 2-way ANOVA with Bonferroni post-hoc testing as applicable, and using nested design when several measurements from the same animal. Associations were investigated by linear regression analysis. Data were considered significantly different when the p-value or adjusted p value (Benjamini- Hochberg when multiple testing required) p_adj_ (or Q) < 0.05.

# Supplemental Tables

## Table S1.

Cardiac magnetic resonance imaging metrices of structural and functional remodeling 1-month after MI

|  | **MI (N_pigs_ = 13)** | **Sham (N_pigs_ = 8)** | ***P* value** |
| --- | --- | --- | --- |
| Infarct size (%) | 18.5 ± 4.5 | 0 ± 0 | <0.0001 |
| Ejection fraction (%) | 40.9 ± 8.1 | 64.5 ± 4.3 | <0.0001 |
| End-diastolic volume (mL) | 174.9 ± 24.8 | 121.2 ± 13.3 | 0.0003 |
| End-systolic volume (mL) | 103.7 ± 23.4 | 49.8 ± 15.5 | <0.0001 |
| Left ventricular mass (g) | 137.6 ± 16.3 | 128.4 ± 19.5 | 0.2992 |
| Heart rate (beats per minute) | 85.2 ± 9.0 | 97.6 ± 18.3 | 0.0657 |
| Left ventricular peak systolic pressure (mmHg) | 98.7 ± 9.9 | 103.8 ± 19.4 | 0.4974 |
| Left ventricular mean diastolic pressure (mmHg) | 5.0 ± 1.9 | 1.5 ± 1.6 | 0.0090 |

## Table S2.

Differentially expressed genes in the BZ compared with remote regions. Highly variable genes are highlighted in yellow.

| Gene | Base Mean | log2Fold Change | lfcSE | stat | pvalue | padj |
| --- | --- | --- | --- | --- | --- | --- |
| QKI | 22.75 | 0.2663 | 0.02132 | 12.49 | 8.376e-36 | 2.552e-34 |
| TIAM2 | 2.198 | 0.3417 | 0.02463 | 13.87 | 9.369e-44 | 3.687e-42 |
| PPP1R14C | 3.794 | 0.2526 | 0.02253 | 11.21 | 3.66e-29 | 8.332e-28 |
| PHACTR2 | 2.685 | 0.2766 | 0.02236 | 12.37 | 3.876e-35 | 1.146e-33 |
| AIG1 | 3.154 | 0.3421 | 0.02521 | 13.57 | 6.014e-42 | 2.268e-40 |
| ARFGEF3 | 3.067 | 0.4253 | 0.02329 | 18.26 | 1.783e-74 | 1.591e-72 |
| MAP3K5 | 32.3 | 0.2661 | 0.02133 | 12.48 | 9.779e-36 | 2.971e-34 |
| MAP7 | 2.337 | 0.2911 | 0.02669 | 10.91 | 1.031e-27 | 2.179e-26 |
| PDE7B | 3.617 | 1.045 | 0.03557 | 29.37 | 1.164e-189 | 6.845e-187 |
| AHI1 | 3.549 | 0.3169 | 0.02412 | 13.14 | 1.959e-39 | 6.894e-38 |
| CCN2 | 1.291 | 0.4884 | 0.02647 | 18.45 | 5.124e-76 | 4.787e-74 |
| ARHGAP18 | 1.689 | 0.4545 | 0.02317 | 19.62 | 1.098e-85 | 1.322e-83 |
| ENSSSCG00000036671 | 3.264 | 0.387 | 0.02144 | 18.05 | 8.21e-73 | 6.954e-71 |
| BACH2 | 5.988 | 0.7401 | 0.02745 | 26.97 | 3.774e-160 | 1.64e-157 |
| KLHL32 | 4.454 | 0.5115 | 0.02591 | 19.74 | 8.969e-87 | 1.149e-84 |
| ASCC3 | 2.918 | 0.261 | 0.02204 | 11.84 | 2.395e-32 | 6.317e-31 |
| FYN | 3.354 | 0.4536 | 0.02516 | 18.03 | 1.123e-72 | 9.43e-71 |
| LAMA4 | 8.968 | 0.266 | 0.02263 | 11.76 | 6.631e-32 | 1.691e-30 |
| HS3ST5 | 4.241 | 0.3495 | 0.0285 | 12.26 | 1.419e-34 | 4.099e-33 |
| TENT5A | 1.699 | 0.2733 | 0.02317 | 11.8 | 4.037e-32 | 1.048e-30 |
| TPM1 | 13.07 | 0.2772 | 0.0223 | 12.43 | 1.804e-35 | 5.398e-34 |
| ENSSSCG00000004598 | 5.144 | 0.3097 | 0.02316 | 13.37 | 8.656e-41 | 3.181e-39 |
| ZNF106 | 6.88 | 0.3232 | 0.02193 | 14.74 | 3.575e-49 | 1.67e-47 |
| SPRED1 | 1.868 | 0.4563 | 0.02291 | 19.91 | 3.022e-88 | 4.028e-86 |
| C15orf41 | 2.379 | 0.3519 | 0.02408 | 14.61 | 2.379e-48 | 1.071e-46 |
| ACTC1 | 2.73 | 0.6445 | 0.02556 | 25.21 | 2.779e-140 | 8.68e-138 |
| CHSY1 | 1.488 | 0.3246 | 0.02459 | 13.2 | 8.889e-40 | 3.151e-38 |
| SERPINB2 | 1.417 | 0.428 | 0.02541 | 16.84 | 1.196e-63 | 7.815e-62 |
| KDSR | 2.886 | 0.2619 | 0.02622 | 9.991 | 1.669e-23 | 2.803e-22 |
| CCBE1 | 5.464 | 0.3971 | 0.01922 | 20.66 | 7.368e-95 | 1.227e-92 |
| ATP8B1 | 5.798 | 0.2842 | 0.02286 | 12.43 | 1.837e-35 | 5.479e-34 |
| ENSSSCG00000033218 | 2.269 | 0.4642 | 0.02032 | 22.85 | 1.595e-115 | 3.321e-113 |
| UACA | 1.875 | 0.2798 | 0.02293 | 12.2 | 3.171e-34 | 9.056e-33 |
| MYO9A | 6.251 | 0.3188 | 0.02129 | 14.98 | 1.027e-50 | 4.959e-49 |
| NID2 | 4.785 | 0.2976 | 0.02367 | 12.57 | 2.989e-36 | 9.193e-35 |
| DAAM1 | 5.772 | 0.3224 | 0.02287 | 14.1 | 3.994e-45 | 1.636e-43 |
| MLLT3 | 2.808 | 0.2646 | 0.02646 | 9.998 | 1.548e-23 | 2.609e-22 |
| NFIB | 10.27 | 0.3368 | 0.02254 | 14.94 | 1.711e-50 | 8.222e-49 |
| SMARCA2 | 2.908 | 0.5338 | 0.02115 | 25.24 | 1.618e-140 | 5.39e-138 |
| FAM189A2 | 5.378 | 0.5176 | 0.0226 | 22.91 | 4.141e-116 | 8.807e-114 |
| MAMDC2 | 1.135 | 0.3578 | 0.02807 | 12.75 | 3.245e-37 | 1.033e-35 |
| ENSSSCG00000005358 | 1.684 | 0.3225 | 0.0232 | 13.9 | 6.026e-44 | 2.381e-42 |
| ZNF462 | 3.591 | 0.3743 | 0.02402 | 15.58 | 9.218e-55 | 4.875e-53 |
| ENSSSCG00000005518 | 2.287 | 0.3784 | 0.02143 | 17.66 | 8.955e-70 | 7.048e-68 |
| NEK6 | 1.157 | 0.2838 | 0.02797 | 10.15 | 3.461e-24 | 6.026e-23 |
| ATP2C1 | 3.41 | 0.2939 | 0.02046 | 14.36 | 8.581e-47 | 3.665e-45 |
| THRB | 32.12 | 0.4268 | 0.02164 | 19.72 | 1.369e-86 | 1.732e-84 |
| LRRFIP2 | 11.37 | 0.3127 | 0.02032 | 15.39 | 1.897e-53 | 9.877e-52 |
| ITGA9 | 2.518 | 0.4017 | 0.02377 | 16.9 | 4.269e-64 | 2.845e-62 |
| TOPAZ1 | 1.789 | 0.3404 | 0.02365 | 14.39 | 5.71e-47 | 2.481e-45 |
| TNNC1 | 2.583 | 0.2725 | 0.02005 | 13.59 | 4.777e-42 | 1.809e-40 |
| SLMAP | 12.03 | 0.2819 | 0.02131 | 13.23 | 6.031e-40 | 2.153e-38 |
| MAGI1 | 1.837 | 0.3045 | 0.02466 | 12.35 | 4.95e-35 | 1.438e-33 |
| ENSSSCG00000036148 | 3.679 | 0.5052 | 0.02887 | 17.5 | 1.5e-68 | 1.127e-66 |
| FRMD4B | 17.72 | 0.4066 | 0.02288 | 17.77 | 1.27e-70 | 1.032e-68 |
| MITF | 15.22 | 0.3206 | 0.02211 | 14.5 | 1.246e-47 | 5.486e-46 |
| RYBP | 2.626 | 0.2676 | 0.0223 | 12 | 3.623e-33 | 9.868e-32 |
| CNTN3 | 1.092 | 0.2534 | 0.02891 | 8.766 | 1.856e-18 | 2.342e-17 |
| LMCD1 | 1.574 | 0.3271 | 0.02519 | 12.99 | 1.484e-38 | 4.928e-37 |
| GRIP2 | 1.586 | 0.3472 | 0.02379 | 14.59 | 3.16e-48 | 1.404e-46 |
| SLC6A6 | 1.785 | 1.167 | 0.02831 | 41.23 | 0 | 0 |
| XPC | 1.492 | 0.341 | 0.02448 | 13.93 | 4.206e-44 | 1.675e-42 |
| SLC9A9 | 2.582 | 0.2567 | 0.02463 | 10.42 | 1.912e-25 | 3.558e-24 |
| GYG1 | 2.197 | 0.2541 | 0.02358 | 10.78 | 4.456e-27 | 9.145e-26 |
| TSC22D2 | 3.902 | 0.3247 | 0.0235 | 13.82 | 1.966e-43 | 7.677e-42 |
| PHC3 | 3.421 | 0.2855 | 0.02087 | 13.68 | 1.342e-42 | 5.161e-41 |
| TNIK | 11.16 | 0.4849 | 0.02404 | 20.17 | 1.821e-90 | 2.494e-88 |
| PLD1 | 9.655 | 0.3632 | 0.02397 | 15.15 | 7.714e-52 | 3.874e-50 |
| FNDC3B | 13.42 | 0.393 | 0.02162 | 18.17 | 8.614e-74 | 7.486e-72 |
| NAALADL2 | 2.388 | 0.2681 | 0.02717 | 9.868 | 5.753e-23 | 9.35e-22 |
| TBL1XR1 | 6.987 | 0.2662 | 0.02234 | 11.92 | 9.326e-33 | 2.499e-31 |
| IGF2BP2 | 1.708 | 0.3448 | 0.02637 | 13.08 | 4.493e-39 | 1.559e-37 |
| ENSSSCG00000040916 | 1.461 | 0.2781 | 0.02491 | 11.16 | 6.228e-29 | 1.396e-27 |
| TP63 | 1.951 | 0.5141 | 0.02795 | 18.39 | 1.574e-75 | 1.417e-73 |
| P3H2 | 1.123 | 0.2821 | 0.02844 | 9.917 | 3.519e-23 | 5.813e-22 |
| ATP13A3 | 3.412 | 0.8682 | 0.02359 | 36.8 | 2.049e-296 | 2.048e-293 |
| ACAP2 | 2.572 | 0.2517 | 0.02135 | 11.79 | 4.594e-32 | 1.187e-30 |
| ENSSSCG00000011851 | 3.185 | 0.2905 | 0.02332 | 12.46 | 1.278e-35 | 3.837e-34 |
| ENSSSCG00000011850 | 1.502 | 0.3342 | 0.02441 | 13.69 | 1.195e-42 | 4.631e-41 |
| STXBP5L | 1.938 | 0.295 | 0.02636 | 11.19 | 4.488e-29 | 1.013e-27 |
| ARHGAP31 | 1.879 | 0.304 | 0.02347 | 12.95 | 2.328e-38 | 7.704e-37 |
| ZBTB20 | 12.37 | 0.2612 | 0.02249 | 11.61 | 3.543e-31 | 8.808e-30 |
| NECTIN3 | 1.525 | 0.2735 | 0.02466 | 11.09 | 1.44e-28 | 3.171e-27 |
| DZIP3 | 4.299 | 0.4749 | 0.02559 | 18.56 | 7.373e-77 | 7.019e-75 |
| CD47 | 2.368 | 0.3201 | 0.02192 | 14.6 | 2.798e-48 | 1.254e-46 |
| CBLB | 7.66 | 0.4426 | 0.02229 | 19.85 | 1.054e-87 | 1.369e-85 |
| ALCAM | 7.314 | 0.2667 | 0.02818 | 9.466 | 2.896e-21 | 4.339e-20 |
| ST3GAL6 | 2.462 | 0.2772 | 0.02224 | 12.46 | 1.165e-35 | 3.508e-34 |
| PROS1 | 4.905 | 0.2524 | 0.02036 | 12.4 | 2.573e-35 | 7.655e-34 |
| ROBO1 | 2.214 | 1.374 | 0.03057 | 44.93 | 0 | 0 |
| ROBO2 | 1.215 | 0.2542 | 0.02747 | 9.253 | 2.182e-20 | 3.076e-19 |
| USP25 | 5.78 | 0.3093 | 0.02162 | 14.3 | 2.069e-46 | 8.689e-45 |
| ENSSSCG00000034679 | 2.104 | 0.4055 | 0.02362 | 17.17 | 4.438e-66 | 3.124e-64 |
| CYYR1 | 1.997 | 0.3455 | 0.0241 | 14.33 | 1.335e-46 | 5.63e-45 |
| RCAN1 | 3.578 | 0.3587 | 0.02502 | 14.34 | 1.251e-46 | 5.299e-45 |
| TTC3 | 2.564 | 0.253 | 0.02152 | 11.76 | 6.249e-32 | 1.601e-30 |
| IL34 | 4.946 | 0.3628 | 0.0241 | 15.05 | 3.21e-51 | 1.58e-49 |
| NDRG4 | 5.459 | 0.3775 | 0.02279 | 16.57 | 1.167e-61 | 7.069e-60 |
| CDH8 | 3.231 | 0.3654 | 0.0287 | 12.73 | 3.876e-37 | 1.23e-35 |
| TERB1 | 1.486 | 0.2633 | 0.02477 | 10.63 | 2.199e-26 | 4.361e-25 |
| TNNT1 | 1.963 | 0.4136 | 0.02485 | 16.65 | 3.191e-62 | 1.957e-60 |
| NPPB | 1.614 | 0.9291 | 0.02767 | 33.58 | 3.759e-247 | 2.89e-244 |
| SRSF10 | 3.253 | 0.4414 | 0.02009 | 21.97 | 5.426e-107 | 1.043e-104 |
| STX12 | 1.93 | 0.3346 | 0.02228 | 15.02 | 5.475e-51 | 2.669e-49 |
| EPB41L3 | 36.43 | 0.364 | 0.02297 | 15.84 | 1.532e-56 | 8.462e-55 |
| GATA6 | 7.788 | 0.2843 | 0.02204 | 12.9 | 4.585e-38 | 1.493e-36 |
| DSC3 | 3.297 | 0.2659 | 0.02032 | 13.08 | 4.091e-39 | 1.425e-37 |
| MAPRE2 | 4.467 | 0.2613 | 0.02462 | 10.61 | 2.571e-26 | 5.038e-25 |
| ENSSSCG00000039133 | 1.428 | 0.3067 | 0.02511 | 12.21 | 2.599e-34 | 7.464e-33 |
| ENSSSCG00000031521 | 1.296 | 0.2921 | 0.02641 | 11.06 | 1.949e-28 | 4.272e-27 |
| PRKACB | 5.447 | 0.2899 | 0.02305 | 12.58 | 2.78e-36 | 8.575e-35 |
| GIPC2 | 7.577 | 0.3701 | 0.02531 | 14.62 | 1.974e-48 | 9.007e-47 |
| DNAJB4 | 8.101 | 0.3014 | 0.02162 | 13.94 | 3.412e-44 | 1.364e-42 |
| ENSSSCG00000003771 | 3.608 | 0.2525 | 0.02015 | 12.53 | 5.13e-36 | 1.573e-34 |
| SLC44A5 | 4.352 | 0.5378 | 0.02561 | 21 | 6.19e-98 | 1.085e-95 |
| PTGER3 | 33.25 | 0.3471 | 0.02494 | 13.92 | 4.89e-44 | 1.94e-42 |
| TCTEX1D1 | 1.902 | 0.2823 | 0.02329 | 12.12 | 8.305e-34 | 2.332e-32 |
| PDE4B | 5.808 | 0.4157 | 0.02372 | 17.52 | 9.527e-69 | 7.269e-67 |
| ROR1 | 6.485 | 0.466 | 0.02792 | 16.69 | 1.57e-62 | 9.807e-61 |
| FAM151A | 1.6 | 0.2549 | 0.02389 | 10.67 | 1.405e-26 | 2.803e-25 |
| ENSSSCG00000038101 | 1.549 | 0.3734 | 0.02818 | 13.25 | 4.291e-40 | 1.537e-38 |
| PIK3R3 | 1.449 | 0.4329 | 0.02496 | 17.35 | 2.032e-67 | 1.494e-65 |
| ENSSSCG00000014540 | 1.406 | 0.2517 | 0.02549 | 9.877 | 5.259e-23 | 8.574e-22 |
| CTNND1 | 3.773 | 0.3886 | 0.0206 | 18.87 | 2.182e-79 | 2.225e-77 |
| CD44 | 2.323 | 0.3073 | 0.02301 | 13.35 | 1.141e-40 | 4.176e-39 |
| ENSSSCG00000035881 | 4.668 | 0.4747 | 0.02741 | 17.32 | 3.368e-67 | 2.457e-65 |
| FSHB | 1.442 | 0.4532 | 0.02458 | 18.44 | 6.32e-76 | 5.849e-74 |
| CSRP3 | 6.189 | 0.3414 | 0.02495 | 13.68 | 1.288e-42 | 4.971e-41 |
| ENSSSCG00000033786 | 2.517 | 0.3576 | 0.02489 | 14.37 | 8.218e-47 | 3.525e-45 |
| MICAL2 | 7.435 | 0.3962 | 0.02191 | 18.08 | 4.318e-73 | 3.688e-71 |
| DKK3 | 1.383 | 0.5764 | 0.02498 | 23.07 | 9.226e-118 | 2.005e-115 |
| ENSSSCG00000013880 | 1.137 | 0.4185 | 0.02779 | 15.06 | 2.993e-51 | 1.481e-49 |
| LHFPL2 | 3.871 | 0.3114 | 0.02337 | 13.33 | 1.628e-40 | 5.916e-39 |
| SSBP2 | 7.079 | 0.3383 | 0.02673 | 12.65 | 1.077e-36 | 3.364e-35 |
| MEF2C | 8.254 | 0.4586 | 0.02216 | 20.69 | 4.06e-95 | 6.879e-93 |
| ENSSSCG00000035571 | 2.529 | 0.3003 | 0.02555 | 11.75 | 6.737e-32 | 1.713e-30 |
| ENSSSCG00000014161 | 2.116 | 0.2516 | 0.02462 | 10.22 | 1.655e-24 | 2.926e-23 |
| SLCO6A1 | 1.546 | 0.3271 | 0.03147 | 10.39 | 2.639e-25 | 4.894e-24 |
| PAM | 10.64 | 0.4828 | 0.02372 | 20.36 | 4.043e-92 | 5.856e-90 |
| EFNA5 | 19.05 | 0.3397 | 0.0252 | 13.48 | 2.019e-41 | 7.559e-40 |
| MCC | 6.776 | 0.2576 | 0.02526 | 10.2 | 2.016e-24 | 3.541e-23 |
| PRR16 | 10.14 | 0.4652 | 0.02466 | 18.86 | 2.272e-79 | 2.294e-77 |
| ZNF608 | 3.027 | 0.2693 | 0.02339 | 11.51 | 1.152e-30 | 2.814e-29 |
| FBN2 | 3.587 | 1.259 | 0.03105 | 40.54 | 0 | 0 |
| ADAMTS19 | 1.118 | 0.3136 | 0.02894 | 10.84 | 2.248e-27 | 4.662e-26 |
| KLHL3 | 2.526 | 0.4318 | 0.02191 | 19.71 | 1.91e-86 | 2.328e-84 |
| MYOT | 5.842 | 0.2763 | 0.02486 | 11.12 | 1.044e-28 | 2.308e-27 |
| DPYSL3 | 4.259 | 0.2545 | 0.02407 | 10.58 | 3.816e-26 | 7.392e-25 |
| PPP3CC | 10.65 | 0.3403 | 0.02034 | 16.73 | 7.938e-63 | 5.053e-61 |
| ADRA1A | 4.496 | 0.3502 | 0.02716 | 12.89 | 4.809e-38 | 1.551e-36 |
| PTK2B | 1.536 | 0.4002 | 0.02406 | 16.63 | 3.979e-62 | 2.425e-60 |
| ADGRD1 | 1.741 | 0.2778 | 0.02423 | 11.47 | 1.939e-30 | 4.692e-29 |
| CLIP1 | 7.872 | 0.2962 | 0.02258 | 13.11 | 2.719e-39 | 9.504e-38 |
| MYL2 | 10.92 | 0.3617 | 0.02063 | 17.53 | 8.541e-69 | 6.567e-67 |
| MTMR3 | 5.998 | 0.4767 | 0.0212 | 22.49 | 5.498e-112 | 1.121e-109 |
| ACTN2 | 29.87 | 0.2851 | 0.02211 | 12.9 | 4.642e-38 | 1.507e-36 |
| SIPA1L2 | 4.684 | 0.2673 | 0.02399 | 11.14 | 7.807e-29 | 1.742e-27 |
| ACTA1 | 4.287 | 0.3658 | 0.02316 | 15.79 | 3.451e-56 | 1.895e-54 |
| FAM13C | 1.766 | 0.3253 | 0.02383 | 13.65 | 1.954e-42 | 7.454e-41 |
| RHOBTB1 | 2.893 | 0.5421 | 0.02458 | 22.06 | 8.533e-108 | 1.706e-105 |
| ARID5B | 2.488 | 0.433 | 0.02322 | 18.65 | 1.321e-77 | 1.294e-75 |
| LRRTM3 | 6.539 | 0.8243 | 0.02294 | 35.94 | 6.644e-283 | 6.037e-280 |
| HERC4 | 6.578 | 0.4126 | 0.02167 | 19.04 | 7.746e-81 | 8.237e-79 |
| STOX1 | 2.744 | 0.4393 | 0.02308 | 19.03 | 9.559e-81 | 1.006e-78 |
| MICU1 | 4.206 | 0.2585 | 0.02186 | 11.83 | 2.828e-32 | 7.418e-31 |
| MCU | 3.235 | 0.4208 | 0.02681 | 15.7 | 1.618e-55 | 8.697e-54 |
| VCL | 6.989 | 0.3967 | 0.02236 | 17.74 | 1.926e-70 | 1.552e-68 |
| RASSF4 | 1.522 | 0.252 | 0.02449 | 10.29 | 7.805e-25 | 1.408e-23 |
| SGMS1 | 5.53 | 0.3763 | 0.02228 | 16.89 | 5.727e-64 | 3.791e-62 |
| ENSSSCG00000010440 | 2.78 | 0.2833 | 0.02197 | 12.89 | 4.883e-38 | 1.569e-36 |
| ANKRD1 | 27.79 | 0.2604 | 0.02673 | 9.741 | 2.019e-22 | 3.203e-21 |
| SH3PXD2A | 2.359 | 0.3493 | 0.02184 | 15.99 | 1.489e-57 | 8.503e-56 |
| NRAP | 12.39 | 0.3002 | 0.02578 | 11.64 | 2.522e-31 | 6.286e-30 |
| INPP5F | 1.965 | 0.4073 | 0.0236 | 17.26 | 9.658e-67 | 6.846e-65 |
| PLEKHA1 | 2.277 | 0.2519 | 0.02103 | 11.98 | 4.567e-33 | 1.24e-31 |
| MGMT | 3.546 | 0.3123 | 0.03037 | 10.29 | 8.186e-25 | 1.474e-23 |
| ENSSSCG00000031528 | 1.824 | 0.6843 | 0.02369 | 28.88 | 1.884e-183 | 1.046e-180 |
| ENSSSCG00000023746 | 2.159 | 0.3023 | 0.02694 | 11.22 | 3.125e-29 | 7.148e-28 |
| R3HDM1 | 8.842 | 0.331 | 0.02231 | 14.83 | 8.811e-50 | 4.194e-48 |
| RAB3GAP1 | 2.736 | 0.2598 | 0.02175 | 11.95 | 6.785e-33 | 1.828e-31 |
| ENSSSCG00000023479 | 9.391 | 0.406 | 0.01988 | 20.42 | 1.048e-92 | 1.611e-90 |
| SLC35F5 | 2.58 | 0.3256 | 0.02088 | 15.6 | 7.773e-55 | 4.132e-53 |
| EPB41L5 | 4.546 | 0.2656 | 0.02098 | 12.66 | 1.038e-36 | 3.252e-35 |
| ENSSSCG00000034187 | 1.698 | 0.4276 | 0.02272 | 18.82 | 5.213e-79 | 5.159e-77 |
| PDLIM3 | 1.592 | 0.3827 | 0.02656 | 14.41 | 4.648e-47 | 2.029e-45 |
| SORBS2 | 106.5 | 0.292 | 0.02299 | 12.7 | 5.869e-37 | 1.845e-35 |
| GALNT13 | 1.242 | 0.5716 | 0.02778 | 20.57 | 4.688e-94 | 7.437e-92 |
| ACVR1 | 4.28 | 0.2766 | 0.02138 | 12.94 | 2.804e-38 | 9.189e-37 |
| RBMS1 | 6.63 | 0.3777 | 0.02256 | 16.74 | 7.009e-63 | 4.491e-61 |
| FIGN | 3.298 | 0.2757 | 0.02477 | 11.13 | 9.031e-29 | 2.01e-27 |
| XIRP2 | 2.571 | 0.4823 | 0.02809 | 17.17 | 4.614e-66 | 3.225e-64 |
| CERS6 | 1.224 | 0.4692 | 0.02738 | 17.14 | 7.985e-66 | 5.542e-64 |
| MAP3K20 | 6.151 | 0.3707 | 0.02326 | 15.94 | 3.589e-57 | 2.015e-55 |
| ITPRID2 | 1.564 | 0.3406 | 0.02402 | 14.18 | 1.232e-45 | 5.066e-44 |
| FRZB | 1.847 | 0.9701 | 0.0273 | 35.54 | 1.212e-276 | 1.01e-273 |
| NCKAP1 | 3.056 | 0.282 | 0.02125 | 13.28 | 3.232e-40 | 1.162e-38 |
| ITGAV | 3.47 | 0.6691 | 0.02361 | 28.35 | 9.416e-177 | 4.482e-174 |
| GULP1 | 12.22 | 0.2736 | 0.02245 | 12.19 | 3.602e-34 | 1.026e-32 |
| MYO1B | 1.161 | 0.4536 | 0.02765 | 16.41 | 1.757e-60 | 1.027e-58 |
| CAVIN2 | 2.038 | 0.3704 | 0.02247 | 16.48 | 4.886e-61 | 2.907e-59 |
| SLC39A10 | 2.267 | 0.375 | 0.02244 | 16.71 | 1.137e-62 | 7.147e-61 |
| HECW2 | 3.04 | 0.652 | 0.02695 | 24.19 | 2.712e-129 | 6.611e-127 |
| PLCL1 | 1.78 | 0.2829 | 0.02399 | 11.79 | 4.261e-32 | 1.103e-30 |
| CLK1 | 2.771 | 0.3081 | 0.01899 | 16.23 | 3.342e-59 | 1.931e-57 |
| STRADB | 9.089 | 0.2844 | 0.02212 | 12.86 | 7.933e-38 | 2.533e-36 |
| ENSSSCG00000036410 | 2.179 | 0.5744 | 0.02021 | 28.43 | 9.441e-178 | 4.718e-175 |
| CYP20A1 | 3.665 | 0.3681 | 0.02163 | 17.02 | 6.211e-65 | 4.252e-63 |
| ENSSSCG00000016119 | 1.994 | 0.4233 | 0.02279 | 18.57 | 5.181e-77 | 5.027e-75 |
| DES | 3.976 | 0.3197 | 0.02315 | 13.81 | 2.177e-43 | 8.465e-42 |
| EPHA4 | 1.251 | 0.4373 | 0.02646 | 16.53 | 2.304e-61 | 1.387e-59 |
| KCNJ13 | 1.514 | 0.3141 | 0.02437 | 12.89 | 5.017e-38 | 1.607e-36 |
| ENSSSCG00000014850 | 1.978 | 0.5208 | 0.02565 | 20.31 | 1.147e-91 | 1.614e-89 |
| CTSC | 9.082 | 0.4092 | 0.02487 | 16.45 | 8.495e-61 | 5.024e-59 |
| ENSSSCG00000038831 | 5.182 | 0.2595 | 0.02207 | 11.76 | 6.43e-32 | 1.644e-30 |
| NAALAD2 | 2.338 | 0.4393 | 0.02255 | 19.48 | 1.634e-84 | 1.921e-82 |
| MAML2 | 3.16 | 0.6303 | 0.02895 | 21.77 | 4.694e-105 | 8.53e-103 |
| CEP126 | 4.482 | 0.6016 | 0.023 | 26.16 | 8.047e-151 | 3.217e-148 |
| PDGFD | 3.433 | 1.275 | 0.02907 | 43.87 | 0 | 0 |
| GRIA4 | 1.095 | 0.2594 | 0.02904 | 8.934 | 4.116e-19 | 5.363e-18 |
| SIK2 | 1.09 | 0.2778 | 0.02884 | 9.631 | 5.892e-22 | 9.145e-21 |
| ENSSSCG00000015027 | 1.345 | 0.2547 | 0.02604 | 9.782 | 1.35e-22 | 2.156e-21 |
| NCAM1 | 7.255 | 0.4702 | 0.02444 | 19.24 | 1.698e-82 | 1.886e-80 |
| CADM1 | 2.553 | 0.3593 | 0.03022 | 11.89 | 1.373e-32 | 3.671e-31 |
| SIK3 | 5.66 | 0.2953 | 0.02262 | 13.05 | 6.151e-39 | 2.113e-37 |
| ARHGEF12 | 5.076 | 0.3037 | 0.02112 | 14.38 | 7.199e-47 | 3.102e-45 |
| GRAMD1B | 5.505 | 0.5453 | 0.02477 | 22.01 | 2.158e-107 | 4.23e-105 |
| SCN3B | 1.862 | 1.022 | 0.02779 | 36.8 | 1.992e-296 | 2.048e-293 |
| ATP2B4 | 6.004 | 0.2596 | 0.02411 | 10.77 | 5.01e-27 | 1.022e-25 |
| ENSSSCG00000028035 | 3.414 | 0.2653 | 0.01977 | 13.42 | 4.599e-41 | 1.702e-39 |
| AKAP9 | 6.053 | 0.2661 | 0.01951 | 13.64 | 2.401e-42 | 9.124e-41 |
| PPP1R9A | 7.537 | 0.2546 | 0.02294 | 11.1 | 1.315e-28 | 2.902e-27 |
| THSD7A | 7.282 | 0.3166 | 0.02619 | 12.09 | 1.219e-33 | 3.405e-32 |
| ETV1 | 1.288 | 0.6378 | 0.02558 | 24.93 | 3.418e-137 | 1.005e-134 |
| BZW2 | 3.426 | 0.3995 | 0.02158 | 18.51 | 1.581e-76 | 1.491e-74 |
| HDAC9 | 15.3 | 0.4268 | 0.02254 | 18.93 | 6.027e-80 | 6.211e-78 |
| ITGB8 | 2.328 | 0.3669 | 0.02487 | 14.75 | 2.895e-49 | 1.359e-47 |
| FAM126A | 4.837 | 0.5286 | 0.02595 | 20.37 | 3.003e-92 | 4.414e-90 |
| CACNA2D1 | 11.88 | 0.3324 | 0.02182 | 15.24 | 2.043e-52 | 1.047e-50 |
| PTPN12 | 2.549 | 0.2626 | 0.02106 | 12.47 | 1.095e-35 | 3.315e-34 |
| TPK1 | 8.508 | 0.2663 | 0.02387 | 11.16 | 6.735e-29 | 1.506e-27 |
| XPR1 | 4.033 | 0.6835 | 0.0278 | 24.59 | 1.79e-133 | 4.588e-131 |
| STX6 | 2.964 | 0.41 | 0.02333 | 17.57 | 3.833e-69 | 2.97e-67 |
| LAMC1 | 7.681 | 0.3359 | 0.0239 | 14.05 | 7.501e-45 | 3.035e-43 |
| NMNAT2 | 2.457 | 0.3323 | 0.02521 | 13.18 | 1.129e-39 | 3.986e-38 |
| LCORL | 4.372 | 0.2685 | 0.02293 | 11.71 | 1.156e-31 | 2.896e-30 |
| APBB2 | 3.176 | 0.257 | 0.02335 | 11.01 | 3.502e-28 | 7.609e-27 |
| LIMCH1 | 5.403 | 0.467 | 0.02415 | 19.34 | 2.69e-83 | 3.055e-81 |
| GABRA4 | 3.877 | 0.5756 | 0.02626 | 21.92 | 1.725e-106 | 3.252e-104 |
| GABRB1 | 9.078 | 0.3815 | 0.02598 | 14.68 | 8.217e-49 | 3.82e-47 |
| GASK1B | 2.672 | 0.4175 | 0.02249 | 18.56 | 6.445e-77 | 6.194e-75 |
| TMEM144 | 3.486 | 0.3099 | 0.02267 | 13.67 | 1.503e-42 | 5.756e-41 |
| TECRL | 27.24 | 0.3863 | 0.02557 | 15.11 | 1.404e-51 | 7.017e-50 |
| ADAMTS3 | 2.571 | 0.4835 | 0.02711 | 17.84 | 3.591e-71 | 2.942e-69 |
| SEPT11 | 1.709 | 0.2664 | 0.0232 | 11.48 | 1.576e-30 | 3.832e-29 |
| FRAS1 | 1.741 | 0.3024 | 0.02633 | 11.48 | 1.605e-30 | 3.893e-29 |
| TRIM2 | 5.533 | 0.2618 | 0.02323 | 11.27 | 1.865e-29 | 4.325e-28 |
| INPP4B | 16.05 | 0.4481 | 0.02202 | 20.36 | 4.124e-92 | 5.889e-90 |
| RNF150 | 14.51 | 0.2873 | 0.02539 | 11.32 | 1.06e-29 | 2.498e-28 |
| MAML3 | 2.849 | 0.5672 | 0.02877 | 19.71 | 1.627e-86 | 2.007e-84 |
| FAT4 | 1.325 | 0.2841 | 0.02645 | 10.74 | 6.415e-27 | 1.303e-25 |
| MYOZ2 | 25.21 | 0.474 | 0.02163 | 21.92 | 1.828e-106 | 3.383e-104 |
| SYNPO2 | 11.83 | 0.4215 | 0.0252 | 16.72 | 8.92e-63 | 5.643e-61 |
| SEC24D | 1.973 | 0.2958 | 0.02264 | 13.06 | 5.284e-39 | 1.821e-37 |
| NDST3 | 1.542 | 0.2717 | 0.02475 | 10.98 | 4.827e-28 | 1.042e-26 |
| NPNT | 1.386 | 0.3799 | 0.02588 | 14.68 | 9.224e-49 | 4.248e-47 |
| ARHGAP24 | 7.703 | 0.4103 | 0.0244 | 16.81 | 2.035e-63 | 1.312e-61 |
| GPAT3 | 3.936 | 0.3075 | 0.02557 | 12.03 | 2.595e-33 | 7.126e-32 |
| ANTXR2 | 3.169 | 0.2714 | 0.02402 | 11.3 | 1.34e-29 | 3.143e-28 |
| HSPB1 | 1.438 | 0.2814 | 0.02511 | 11.21 | 3.812e-29 | 8.658e-28 |
| HIP1 | 2.054 | 0.2752 | 0.02116 | 13.01 | 1.144e-38 | 3.836e-37 |
| RABGEF1 | 1.726 | 0.3094 | 0.02296 | 13.47 | 2.259e-41 | 8.424e-40 |
| ENSSSCG00000035273 | 1.358 | 0.5149 | 0.02695 | 19.1 | 2.426e-81 | 2.607e-79 |
| TACR1 | 1.653 | 0.2681 | 0.02655 | 10.09 | 5.823e-24 | 9.932e-23 |
| SPRED2 | 1.436 | 0.5072 | 0.02463 | 20.59 | 3.323e-94 | 5.356e-92 |
| CCDC85A | 7.756 | 0.31 | 0.02854 | 10.86 | 1.717e-27 | 3.582e-26 |
| RTN4 | 2.793 | 0.2984 | 0.02511 | 11.88 | 1.435e-32 | 3.825e-31 |
| ENSSSCG00000035426 | 2.97 | 0.5392 | 0.02296 | 23.49 | 5.716e-122 | 1.298e-119 |
| PREPL | 2.224 | 0.3954 | 0.02249 | 17.58 | 3.523e-69 | 2.751e-67 |
| ENSSSCG00000008451 | 2.477 | 0.2658 | 0.02132 | 12.47 | 1.13e-35 | 3.413e-34 |
| EML4 | 2.009 | 0.2624 | 0.02222 | 11.81 | 3.645e-32 | 9.487e-31 |
| ENSSSCG00000008496 | 1.623 | 0.25 | 0.02385 | 10.48 | 1.02e-25 | 1.931e-24 |
| LTBP1 | 1.967 | 1.353 | 0.02787 | 48.54 | 0 | 0 |
| TRIM54 | 3.55 | 0.2575 | 0.02226 | 11.57 | 6.036e-31 | 1.497e-29 |
| EFR3B | 1.639 | 0.2905 | 0.02352 | 12.35 | 4.732e-35 | 1.383e-33 |
| ROCK2 | 4.864 | 0.3617 | 0.02265 | 15.97 | 2.216e-57 | 1.258e-55 |
| MBOAT2 | 2.55 | 0.3039 | 0.02258 | 13.46 | 2.784e-41 | 1.034e-39 |
| MTSS1 | 3.75 | 0.2653 | 0.02194 | 12.09 | 1.197e-33 | 3.352e-32 |
| ENSSSCG00000034048 | 2.287 | 0.6462 | 0.02055 | 31.45 | 4.521e-217 | 3.227e-214 |
| TMEM65 | 9.547 | 0.448 | 0.02253 | 19.89 | 5.238e-88 | 6.889e-86 |
| EXT1 | 12.95 | 0.4704 | 0.02327 | 20.21 | 7.379e-91 | 1.024e-88 |
| ENSSSCG00000006008 | 1.311 | 0.3186 | 0.02616 | 12.18 | 4.196e-34 | 1.191e-32 |
| ANGPT1 | 1.168 | 0.4948 | 0.02724 | 18.16 | 9.962e-74 | 8.584e-72 |
| YWHAZ | 4.103 | 0.4431 | 0.02312 | 19.17 | 7.246e-82 | 7.959e-80 |
| PTDSS1 | 7.451 | 0.3315 | 0.02318 | 14.3 | 2.112e-46 | 8.831e-45 |
| RUNX1T1 | 7.468 | 0.3433 | 0.02342 | 14.66 | 1.159e-48 | 5.313e-47 |
| CPNE3 | 11.61 | 0.2761 | 0.02064 | 13.38 | 8.169e-41 | 3.013e-39 |
| PAG1 | 4.47 | 0.3689 | 0.02495 | 14.79 | 1.763e-49 | 8.312e-48 |
| ZC2HC1A | 2.424 | 0.2803 | 0.02108 | 13.3 | 2.385e-40 | 8.636e-39 |
| CSPP1 | 2.08 | 0.2783 | 0.02228 | 12.49 | 8.235e-36 | 2.517e-34 |
| PDE7A | 8.027 | 0.2738 | 0.02214 | 12.37 | 3.895e-35 | 1.148e-33 |
| KIFAP3 | 2.011 | 0.3092 | 0.02164 | 14.29 | 2.471e-46 | 1.029e-44 |
| ATP1B1 | 5.35 | 0.3874 | 0.02106 | 18.39 | 1.479e-75 | 1.344e-73 |
| DPT | 1.458 | 0.2641 | 0.02499 | 10.57 | 4.149e-26 | 8.021e-25 |
| NES | 1.163 | 0.2681 | 0.02796 | 9.59 | 8.767e-22 | 1.346e-20 |
| ARHGEF2 | 2.746 | 0.2776 | 0.02145 | 12.94 | 2.676e-38 | 8.798e-37 |
| RNF115 | 3.053 | 0.3473 | 0.02233 | 15.56 | 1.406e-54 | 7.357e-53 |
| MAGI3 | 2.213 | 0.2505 | 0.02512 | 9.974 | 1.973e-23 | 3.292e-22 |
| CDC14A | 2.099 | 0.7965 | 0.02553 | 31.2 | 1.21e-213 | 8.066e-211 |
| PALMD | 7.166 | 0.4971 | 0.02434 | 20.42 | 1.075e-92 | 1.628e-90 |
| MID1 | 1.22 | 0.2858 | 0.02726 | 10.49 | 9.834e-26 | 1.865e-24 |
| CNKSR2 | 3.357 | 0.4767 | 0.0249 | 19.14 | 1.102e-81 | 1.197e-79 |
| PHEX | 2.955 | 0.3681 | 0.02276 | 16.17 | 7.644e-59 | 4.391e-57 |
| NONO | 2.66 | 0.2673 | 0.02053 | 13.02 | 9.038e-39 | 3.073e-37 |
| ENSSSCG00000012403 | 3.378 | 0.3964 | 0.01932 | 20.52 | 1.548e-93 | 2.418e-91 |
| ATRX | 5.214 | 0.2583 | 0.02091 | 12.35 | 4.832e-35 | 1.408e-33 |
| ENSSSCG00000035722 | 1.746 | 0.3907 | 0.02261 | 17.28 | 6.954e-67 | 5.037e-65 |
| COL4A5 | 2.974 | 0.4389 | 0.02317 | 18.94 | 5.228e-80 | 5.443e-78 |
| AMMECR1 | 1.948 | 0.337 | 0.02164 | 15.57 | 1.084e-54 | 5.701e-53 |
| PLS3 | 1.865 | 0.344 | 0.02377 | 14.47 | 1.792e-47 | 7.854e-46 |
| ENSSSCG00000025028 | 3.128 | 0.3314 | 0.02195 | 15.1 | 1.613e-51 | 8.021e-50 |
| ENSSSCG00000012700 | 3.064 | 0.26 | 0.02292 | 11.34 | 8.102e-30 | 1.928e-28 |
| ATP11C | 3.004 | 0.4346 | 0.02433 | 17.87 | 2.157e-71 | 1.781e-69 |
| PHACTR1 | 7.254 | 0.3808 | 0.02723 | 13.99 | 1.913e-44 | 7.681e-43 |
| KIF13A | 7.194 | 0.3029 | 0.02073 | 14.61 | 2.319e-48 | 1.049e-46 |
| RNF144B | 3.164 | 0.7196 | 0.02846 | 25.29 | 4.509e-141 | 1.554e-138 |
| ENSSSCG00000037166 | 1.526 | 0.2589 | 0.02661 | 9.728 | 2.301e-22 | 3.638e-21 |
| MBOAT1 | 2.058 | 0.3622 | 0.02378 | 15.23 | 2.218e-52 | 1.131e-50 |
| ENSSSCG00000001097 | 2.88 | 0.2625 | 0.0227 | 11.57 | 6.197e-31 | 1.533e-29 |
| ENSSSCG00000030197 | 1.833 | 0.5682 | 0.02357 | 24.11 | 2.129e-128 | 5.067e-126 |
| HDGFL3 | 2.37 | 0.2716 | 0.02196 | 12.37 | 3.909e-35 | 1.149e-33 |
| BTBD1 | 4.036 | 0.314 | 0.02182 | 14.39 | 6.131e-47 | 2.653e-45 |
| PEAK1 | 4.857 | 0.6256 | 0.02466 | 25.37 | 5.594e-142 | 2.071e-139 |
| PTPN9 | 2.444 | 0.2628 | 0.02203 | 11.93 | 8.497e-33 | 2.283e-31 |
| STXBP6 | 8.894 | 0.4358 | 0.02546 | 17.12 | 1.031e-65 | 7.105e-64 |
| ENSSSCG00000031822 | 2.021 | 0.51 | 0.02068 | 24.66 | 2.658e-134 | 6.991e-132 |
| SLCO3A1 | 13.95 | 0.3682 | 0.02618 | 14.07 | 6.188e-45 | 2.514e-43 |
| ENSSSCG00000002259 | 6.069 | 0.3044 | 0.02335 | 13.04 | 7.462e-39 | 2.545e-37 |
| SIPA1L1 | 2.172 | 0.2811 | 0.02269 | 12.39 | 2.978e-35 | 8.831e-34 |
| RGS6 | 2.641 | 1.863 | 0.02941 | 63.32 | 0 | 0 |
| NRXN3 | 1.205 | 0.501 | 0.03008 | 16.65 | 2.785e-62 | 1.719e-60 |
| ENSSSCG00000021315 | 1.457 | 0.7373 | 0.03033 | 24.31 | 1.585e-130 | 3.96e-128 |
| FLRT2 | 1.079 | 0.2709 | 0.02904 | 9.328 | 1.074e-20 | 1.545e-19 |
| C12orf75 | 1.317 | 0.2726 | 0.02628 | 10.37 | 3.292e-25 | 6.049e-24 |
| TEX49 | 2.918 | 0.4996 | 0.01981 | 25.22 | 2.156e-140 | 6.95e-138 |
| RASSF3 | 4.177 | 0.4382 | 0.02576 | 17.01 | 7.162e-65 | 4.87e-63 |
| ENSSSCG00000034610 | 1.206 | 0.4432 | 0.02699 | 16.42 | 1.298e-60 | 7.631e-59 |
| FAR2 | 4.083 | 0.2686 | 0.02607 | 10.3 | 6.81e-25 | 1.238e-23 |
| PPFIBP1 | 8.347 | 0.515 | 0.02211 | 23.29 | 5.13e-120 | 1.139e-117 |
| STK38L | 6.934 | 0.2764 | 0.02244 | 12.32 | 7.131e-35 | 2.066e-33 |
| RASSF8 | 4.046 | 0.312 | 0.02466 | 12.65 | 1.116e-36 | 3.476e-35 |
| LMNTD1 | 7.724 | 0.2659 | 0.03015 | 8.819 | 1.157e-18 | 1.473e-17 |
| LDHB | 4.521 | 0.3579 | 0.02048 | 17.48 | 2.197e-68 | 1.639e-66 |
| ENSSSCG00000036623 | 1.996 | 0.4078 | 0.02113 | 19.3 | 5.386e-83 | 6.049e-81 |
| MGST1 | 1.744 | 0.3615 | 0.02267 | 15.95 | 2.857e-57 | 1.613e-55 |
| RERG | 1.115 | 0.3626 | 0.02851 | 12.72 | 4.525e-37 | 1.427e-35 |
| OLR1 | 1.629 | 0.3313 | 0.02368 | 13.99 | 1.766e-44 | 7.118e-43 |
| CCND2 | 1.816 | 0.2543 | 0.02268 | 11.21 | 3.528e-29 | 8.051e-28 |
| DCP1B | 2.035 | 0.3819 | 0.02099 | 18.2 | 5.512e-74 | 4.833e-72 |
| SLC38A1 | 2.313 | 0.3046 | 0.02341 | 13.01 | 1.069e-38 | 3.599e-37 |
| SLC38A2 | 2.026 | 0.2824 | 0.0215 | 13.13 | 2.16e-39 | 7.575e-38 |
| FGD6 | 2.225 | 0.5636 | 0.02363 | 23.86 | 8.653e-126 | 2.011e-123 |
| TMTC2 | 3.268 | 0.3229 | 0.02391 | 13.51 | 1.457e-41 | 5.474e-40 |
| CDH18 | 1.462 | 0.5436 | 0.02555 | 21.28 | 1.878e-100 | 3.352e-98 |
| CDH9 | 1.639 | 0.4537 | 0.02569 | 17.66 | 8.666e-70 | 6.874e-68 |
| PLPP1 | 6.426 | 0.345 | 0.0242 | 14.26 | 4.127e-46 | 1.712e-44 |
| IL6ST | 3.701 | 0.2669 | 0.0211 | 12.65 | 1.165e-36 | 3.617e-35 |
| RNF180 | 6.207 | 0.2844 | 0.0264 | 10.77 | 4.6e-27 | 9.422e-26 |
| RGS7BP | 2.913 | 0.2539 | 0.02487 | 10.21 | 1.779e-24 | 3.136e-23 |
| ADAMTS6 | 6.343 | 0.4845 | 0.02492 | 19.44 | 3.48e-84 | 4.045e-82 |
| ERBIN | 4.384 | 0.3137 | 0.02138 | 14.68 | 9.141e-49 | 4.23e-47 |
| MAST4 | 7.032 | 0.5888 | 0.02364 | 24.91 | 5.787e-137 | 1.653e-134 |
| MAP1B | 2.47 | 0.3017 | 0.02576 | 11.71 | 1.113e-31 | 2.795e-30 |
| TNPO1 | 3.276 | 0.2554 | 0.02007 | 12.72 | 4.405e-37 | 1.393e-35 |
| SH3PXD2B | 2.366 | 0.3409 | 0.02182 | 15.62 | 5.165e-55 | 2.761e-53 |
| STK10 | 1.355 | 0.3736 | 0.02559 | 14.6 | 2.883e-48 | 1.287e-46 |
| WWC1 | 1.225 | 0.4506 | 0.02673 | 16.86 | 9.266e-64 | 6.093e-62 |
| CYFIP2 | 3.557 | 0.7805 | 0.02533 | 30.81 | 2.064e-208 | 1.289e-205 |
| GRIA1 | 12.24 | 0.7124 | 0.02827 | 25.2 | 4.167e-140 | 1.262e-137 |
| ENSSSCG00000009341 | 5.248 | 0.3056 | 0.02149 | 14.22 | 6.625e-46 | 2.736e-44 |
| PDS5B | 3.167 | 0.2595 | 0.02186 | 11.87 | 1.6e-32 | 4.241e-31 |
| ENSSSCG00000038414 | 2.379 | 0.2757 | 0.02343 | 11.77 | 5.777e-32 | 1.484e-30 |
| LHFPL6 | 2.901 | 0.463 | 0.03022 | 15.32 | 5.489e-53 | 2.828e-51 |
| TPT1 | 1.524 | 0.2693 | 0.02444 | 11.02 | 3.086e-28 | 6.72e-27 |
| TSC22D1 | 2.262 | 0.3166 | 0.02125 | 14.9 | 3.135e-50 | 1.499e-48 |
| LMO7 | 13.64 | 0.2565 | 0.01989 | 12.9 | 4.727e-38 | 1.529e-36 |
| TGFB2 | 1.835 | 0.2609 | 0.02455 | 10.63 | 2.226e-26 | 4.397e-25 |
| ENSSSCG00000038646 | 2.108 | 0.4174 | 0.0246 | 16.96 | 1.527e-64 | 1.025e-62 |
| CDC42BPA | 4.967 | 0.304 | 0.02096 | 14.5 | 1.186e-47 | 5.246e-46 |
| ENSSSCG00000027557 | 4.465 | 0.3977 | 0.02302 | 17.28 | 7.092e-67 | 5.099e-65 |
| FANCC | 1.517 | 0.2604 | 0.02452 | 10.62 | 2.392e-26 | 4.707e-25 |
| AOPEP | 20.65 | 0.2525 | 0.02102 | 12.01 | 3.023e-33 | 8.256e-32 |
| FRMD4A | 1.717 | 0.908 | 0.02385 | 38.06 | 0 | 0 |
| ARHGAP21 | 3.767 | 0.3337 | 0.02195 | 15.2 | 3.427e-52 | 1.739e-50 |
| NEBL | 129.8 | 0.3701 | 0.02012 | 18.39 | 1.448e-75 | 1.327e-73 |
| ITGB1 | 3.749 | 0.2755 | 0.02112 | 13.04 | 7.008e-39 | 2.399e-37 |
| DLC1 | 64.24 | 0.342 | 0.02178 | 15.71 | 1.359e-55 | 7.344e-54 |
| TUSC3 | 2.995 | 0.3604 | 0.02285 | 15.77 | 4.976e-56 | 2.718e-54 |
| VDAC3 | 2.25 | 0.2634 | 0.02023 | 13.02 | 9.115e-39 | 3.088e-37 |
| DSTN | 1.88 | 0.286 | 0.02421 | 11.81 | 3.311e-32 | 8.664e-31 |
| C20orf194 | 2.573 | 0.2781 | 0.02306 | 12.06 | 1.732e-33 | 4.795e-32 |
| DLGAP4 | 1.961 | 0.6435 | 0.02436 | 26.41 | 9.416e-154 | 3.922e-151 |
| TGM2 | 2.284 | 0.4692 | 0.02578 | 18.2 | 4.893e-74 | 4.328e-72 |
| NCOA3 | 2.942 | 0.2514 | 0.02181 | 11.52 | 9.897e-31 | 2.424e-29 |
| SULF2 | 1.393 | 0.399 | 0.02517 | 15.85 | 1.429e-56 | 7.937e-55 |
| TANC2 | 2.69 | 0.4583 | 0.02352 | 19.49 | 1.432e-84 | 1.704e-82 |
| ATP6V0A1 | 2.234 | 0.3136 | 0.02145 | 14.62 | 2.153e-48 | 9.779e-47 |
| NF1 | 5.325 | 0.2838 | 0.0215 | 13.2 | 8.796e-40 | 3.129e-38 |
| NLK | 5.104 | 0.3055 | 0.02346 | 13.02 | 9.565e-39 | 3.23e-37 |
| NXN | 1.565 | 0.3165 | 0.02588 | 12.23 | 2.167e-34 | 6.243e-33 |
| ENSSSCG00000038144 | 1.631 | 0.3136 | 0.02424 | 12.94 | 2.849e-38 | 9.307e-37 |
| CREB3L2 | 2.074 | 0.2799 | 0.02337 | 11.98 | 4.598e-33 | 1.246e-31 |
| CHRM2 | 16.07 | 0.6364 | 0.02572 | 24.74 | 3.965e-135 | 1.101e-132 |
| CALD1 | 7.577 | 0.4119 | 0.0247 | 16.68 | 1.943e-62 | 1.206e-60 |
| ENSSSCG00000035751 | 3.004 | 0.5116 | 0.02009 | 25.47 | 4.257e-143 | 1.637e-140 |
| MKLN1 | 5.123 | 0.39 | 0.022 | 17.73 | 2.453e-70 | 1.961e-68 |
| UBE2H | 9.333 | 0.3412 | 0.0222 | 15.37 | 2.77e-53 | 1.434e-51 |
| STRIP2 | 3.011 | 0.3305 | 0.022 | 15.02 | 5.067e-51 | 2.482e-49 |
| CAV1 | 2.061 | 0.4638 | 0.02319 | 20 | 5.146e-89 | 6.951e-87 |
| MDFIC | 1.259 | 0.2932 | 0.02679 | 10.94 | 7.094e-28 | 1.518e-26 |
| DOCK4 | 4.256 | 0.6481 | 0.02562 | 25.29 | 3.783e-141 | 1.35e-138 |
| SEPT7 | 2.167 | 0.2618 | 0.0208 | 12.59 | 2.49e-36 | 7.706e-35 |
| DPY19L1 | 1.923 | 0.2583 | 0.02246 | 11.5 | 1.327e-30 | 3.235e-29 |
| NPSR1 | 3.679 | 0.2735 | 0.03436 | 7.96 | 1.72e-15 | 1.782e-14 |
| CREB5 | 6.812 | 0.5862 | 0.02824 | 20.76 | 1.027e-95 | 1.77e-93 |
| KCNT2 | 1.615 | 1.08 | 0.02515 | 42.94 | 0 | 0 |
| UST | 3.386 | -0.2759 | 0.02627 | -10.5 | 8.366e-26 | 1.596e-24 |
| NHSL1 | 3.45 | -0.2835 | 0.024 | -11.81 | 3.447e-32 | 8.997e-31 |
| EYA4 | 2.582 | -0.4928 | 0.02854 | -17.27 | 8.431e-67 | 6.019e-65 |
| PTPRK | 12.94 | -0.4939 | 0.02505 | -19.72 | 1.539e-86 | 1.923e-84 |
| HEY2 | 1.97 | -0.2696 | 0.0238 | -11.33 | 9.711e-30 | 2.305e-28 |
| DAPK2 | 1.768 | -0.4134 | 0.02538 | -16.29 | 1.166e-59 | 6.773e-58 |
| ADAMTSL1 | 9.748 | -0.2901 | 0.0264 | -10.99 | 4.331e-28 | 9.37e-27 |
| TPM2 | 14.47 | -0.3544 | 0.02104 | -16.84 | 1.278e-63 | 8.294e-62 |
| TSEN2 | 1.825 | -0.2825 | 0.02732 | -10.34 | 4.534e-25 | 8.284e-24 |
| GREB1L | 1.705 | -0.2556 | 0.02527 | -10.11 | 4.783e-24 | 8.2e-23 |
| OSBPL5 | 2.286 | -0.4138 | 0.02369 | -17.47 | 2.647e-68 | 1.96e-66 |
| MACROD1 | 3.228 | -0.3977 | 0.02341 | -16.99 | 9.5e-65 | 6.416e-63 |
| ENSSSCG00000036669 | 4.441 | -0.3848 | 0.02683 | -14.34 | 1.165e-46 | 4.955e-45 |
| MYBPC3 | 14.79 | -0.2621 | 0.02018 | -12.99 | 1.441e-38 | 4.8e-37 |
| LDLRAD3 | 2.702 | -0.2558 | 0.02582 | -9.906 | 3.913e-23 | 6.443e-22 |
| LGR4 | 2.459 | -0.2933 | 0.02539 | -11.55 | 7.512e-31 | 1.849e-29 |
| NREP | 3.121 | -0.2677 | 0.0268 | -9.989 | 1.703e-23 | 2.856e-22 |
| FGF1 | 5.449 | -0.26 | 0.0254 | -10.23 | 1.394e-24 | 2.484e-23 |
| ARHGAP26 | 7.178 | -0.3715 | 0.02357 | -15.77 | 5.398e-56 | 2.932e-54 |
| PPARGC1B | 3.681 | -0.2939 | 0.02204 | -13.34 | 1.425e-40 | 5.199e-39 |
| CAMK2A | 6.192 | -0.5457 | 0.02206 | -24.74 | 4.433e-135 | 1.197e-132 |
| LPL | 7.154 | -0.2529 | 0.02363 | -10.7 | 9.702e-27 | 1.951e-25 |
| LZTS1 | 1.883 | -0.3013 | 0.025 | -12.05 | 1.924e-33 | 5.298e-32 |
| RYR2 | 154.3 | -0.3818 | 0.02182 | -17.5 | 1.413e-68 | 1.07e-66 |
| NID1 | 5.006 | -0.2967 | 0.02469 | -12.02 | 2.812e-33 | 7.7e-32 |
| PRKG1 | 5.726 | -0.3463 | 0.03363 | -10.3 | 7.23e-25 | 1.311e-23 |
| ZBTB16 | 10.95 | -0.284 | 0.02537 | -11.19 | 4.379e-29 | 9.901e-28 |
| KCNJ5 | 1.292 | -0.283 | 0.02885 | -9.807 | 1.05e-22 | 1.685e-21 |
| SLC41A1 | 4.738 | -0.35 | 0.02308 | -15.16 | 6.21e-52 | 3.135e-50 |
| CDK6 | 6.754 | -0.3046 | 0.02548 | -11.95 | 6.224e-33 | 1.681e-31 |
| SYT14 | 1.74 | -0.2782 | 0.02912 | -9.555 | 1.235e-21 | 1.882e-20 |
| KIAA1211 | 1.876 | -0.3295 | 0.02699 | -12.21 | 2.708e-34 | 7.755e-33 |
| ENSSSCG00000036217 | 1.386 | -0.4068 | 0.02892 | -14.07 | 5.914e-45 | 2.413e-43 |
| SRL | 8.898 | -0.3255 | 0.01971 | -16.52 | 2.802e-61 | 1.677e-59 |
| MAP4K3 | 2.45 | -0.5329 | 0.02585 | -20.61 | 2.099e-94 | 3.44e-92 |
| PTP4A3 | 2.44 | -0.2562 | 0.02106 | -12.17 | 4.508e-34 | 1.276e-32 |
| COL14A1 | 2.382 | -0.3369 | 0.02431 | -13.86 | 1.147e-43 | 4.494e-42 |
| TRPS1 | 1.56 | -0.4244 | 0.02864 | -14.82 | 1.101e-49 | 5.216e-48 |
| FKBP5 | 3.247 | -0.318 | 0.02958 | -10.75 | 5.972e-27 | 1.216e-25 |
| VEGFA | 4.773 | -0.3888 | 0.02158 | -18.02 | 1.36e-72 | 1.133e-70 |
| PLA2G7 | 4.876 | -0.2794 | 0.02487 | -11.24 | 2.686e-29 | 6.187e-28 |
| ACO2 | 7.758 | -0.2855 | 0.02197 | -13 | 1.296e-38 | 4.332e-37 |
| SUN2 | 2.714 | -0.2546 | 0.02588 | -9.837 | 7.794e-23 | 1.257e-21 |
| ITGA5 | 2.311 | -0.2613 | 0.02225 | -11.74 | 7.779e-32 | 1.968e-30 |
| ADAMTS20 | 2.2 | -0.9973 | 0.03502 | -28.48 | 2.339e-178 | 1.23e-175 |
| STC2 | 1.82 | -0.2993 | 0.0248 | -12.07 | 1.518e-33 | 4.225e-32 |
| TENM2 | 2.159 | -0.5734 | 0.02951 | -19.43 | 4.314e-84 | 4.956e-82 |
| ENSSSCG00000017019 | 2.503 | -0.6231 | 0.03053 | -20.41 | 1.328e-92 | 1.981e-90 |
| MARC2 | 2.426 | -0.2883 | 0.02205 | -13.07 | 4.773e-39 | 1.651e-37 |
| KIF26B | 11.06 | -0.8484 | 0.03107 | -27.31 | 3.435e-164 | 1.561e-161 |
| DAPK1 | 3.218 | -0.3042 | 0.02774 | -10.97 | 5.626e-28 | 1.209e-26 |
| RIN2 | 4.167 | -0.3371 | 0.02539 | -13.28 | 3.138e-40 | 1.132e-38 |
| ACSS1 | 2.096 | -0.4764 | 0.02528 | -18.84 | 3.31e-79 | 3.308e-77 |
| KCNH2 | 1.704 | -0.328 | 0.02534 | -12.94 | 2.606e-38 | 8.597e-37 |
| TSPAN12 | 17.88 | -0.2571 | 0.0235 | -10.94 | 7.323e-28 | 1.564e-26 |
| ADCYAP1R1 | 1.612 | -0.4219 | 0.02654 | -15.9 | 6.521e-57 | 3.641e-55 |
| ENSSSCG00000032599 | 1.595 | -0.2941 | 0.02644 | -11.12 | 9.768e-29 | 2.165e-27 |
| ENSSSCG00000032395 | 5.477 | -0.506 | 0.0337 | -15.02 | 5.816e-51 | 2.822e-49 |

## Figure S1


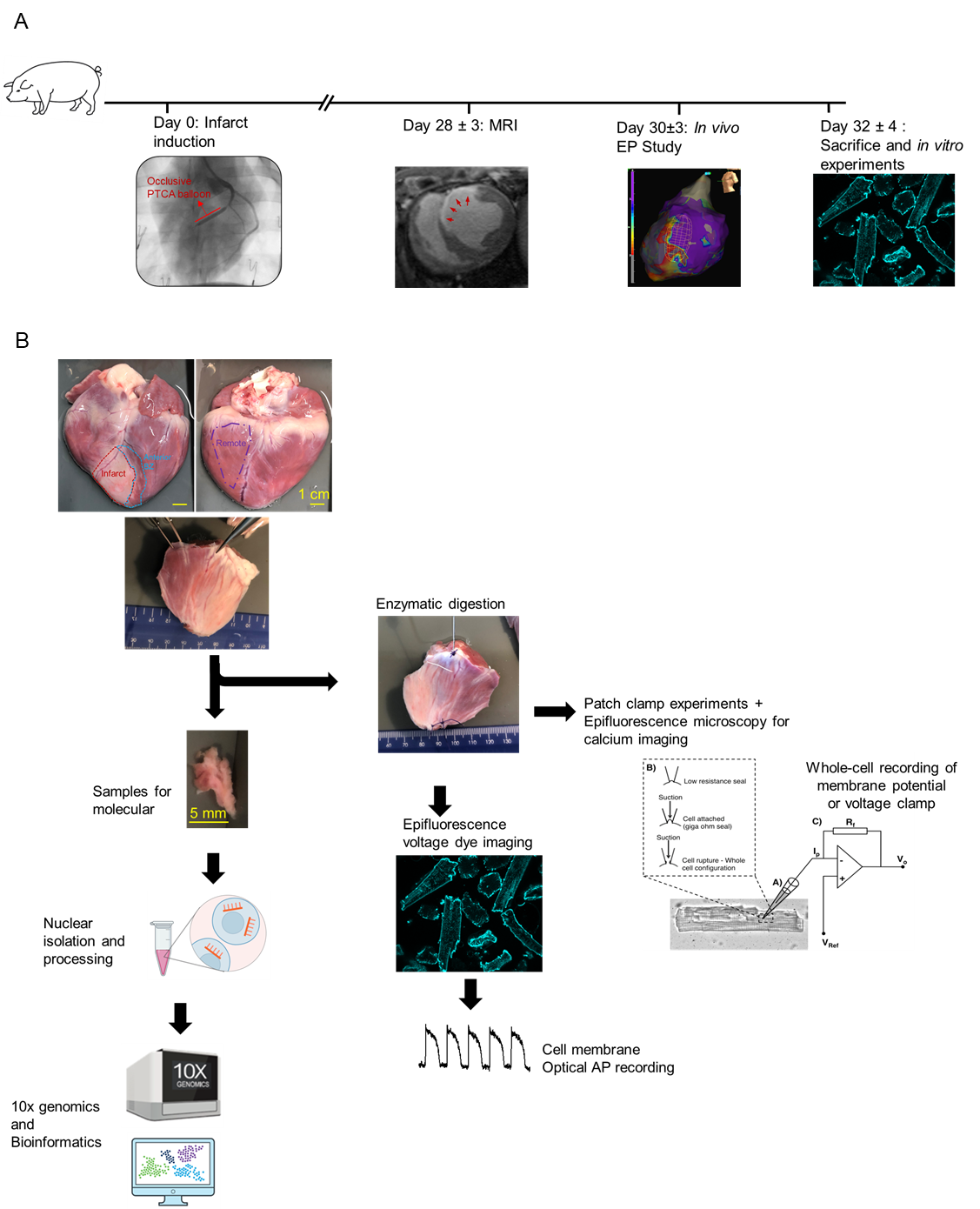


**Figure S1. Experimental design and tissue sampling.**

**A,** Timeline of the experimental protocol.

**B,** Anterior (left) and posterior view (right) of the heart, and illustration of the workflow for cellular and molecular studies.

## Figure S2


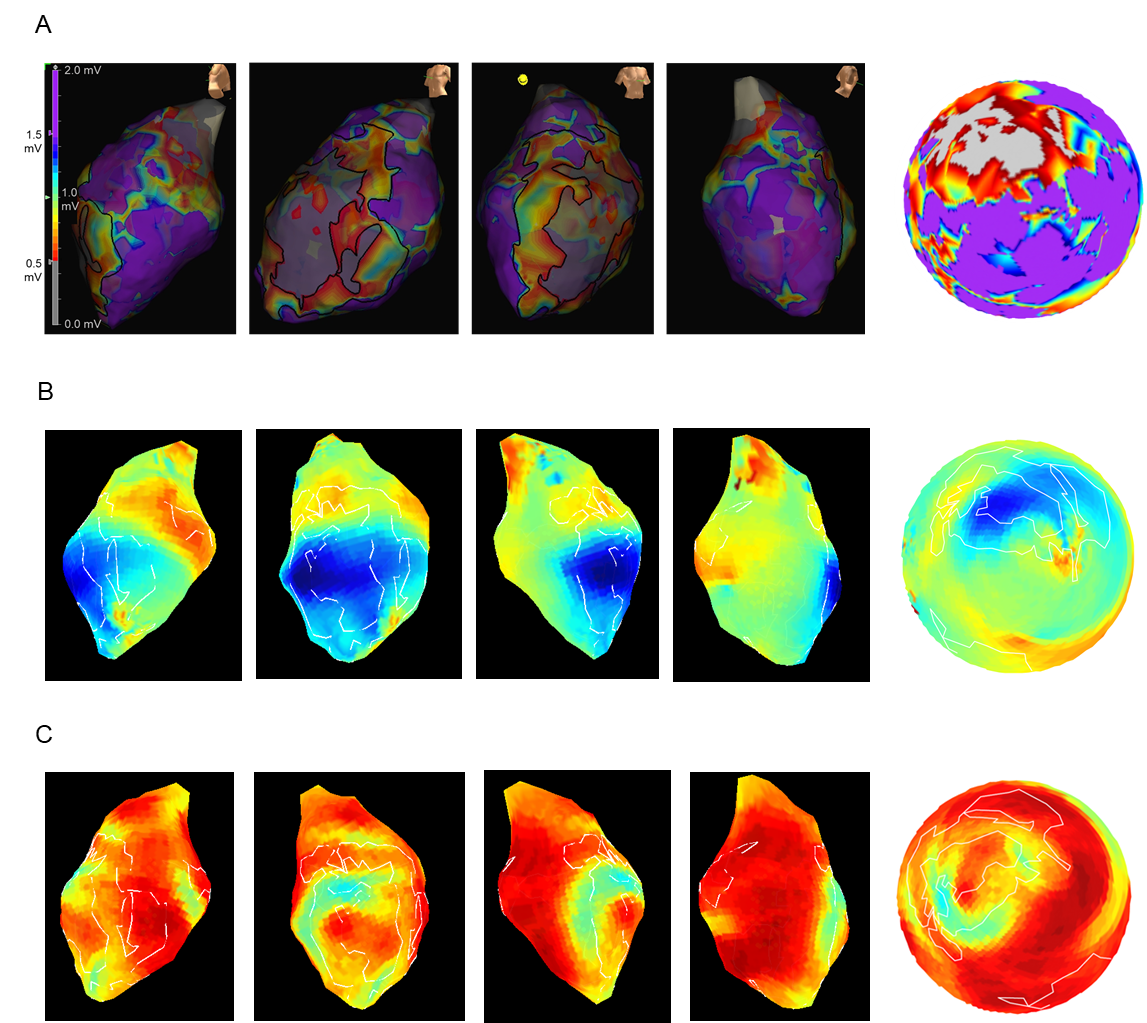


**Figure S2. Assessing regional heterogeneity of local activation-recovery interval (ARI) *in vivo* after MI.**

**A,** Example of lateral, left-anterior oblique, right-anterior oblique and posterior views of electroanatomical mapping model generated to define the BZ as the region of bipolar voltage 0.5-1.5 mV. This model is visualized in 2D as a polar map (right).
**B**, Corresponding non-contact mapping model illustrating the local ARI and translation to a polar map.

**C,** Local ARI heterogeneity quantified as the standard deviation of the electrogram ARIs in a 1 cm radius for each electrogram.

## Figure S3


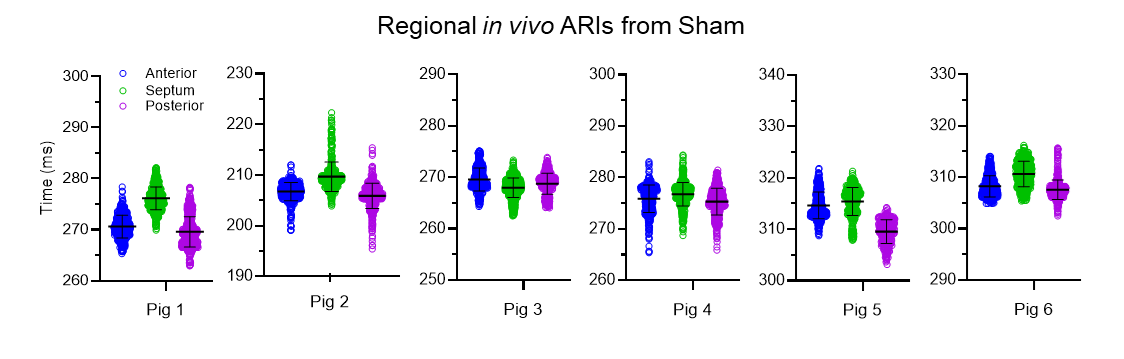


**Figure S3. Quantification of *in vivo* heterogeneity of repolarization.**

Pooled data on ARIs of different regions *in vivo* from Sham (N_pigs_ = 6, n_ARI_ = 321-568/region).

## Figure S4


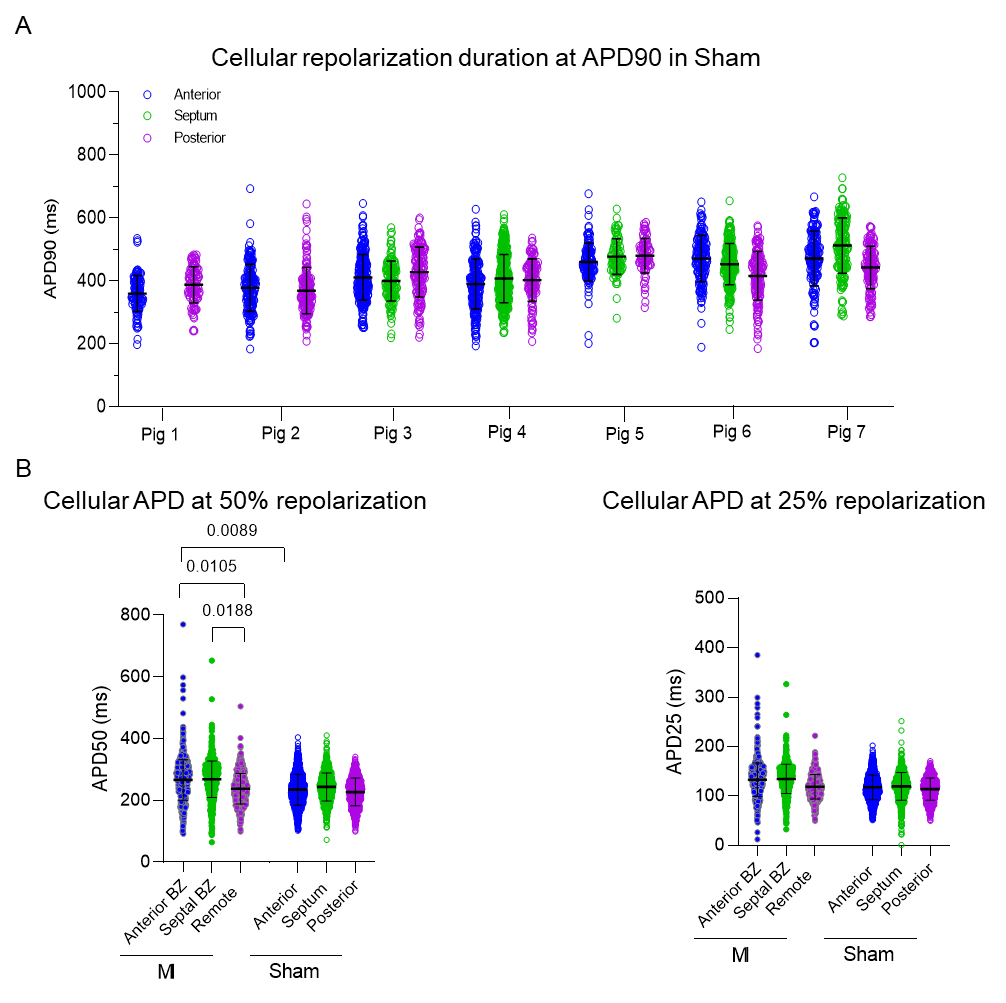


**Figure S4. Heterogeneity of early repolarization after MI.**

**A,** Pooled individual cellular APD90 of populations of isolated cells from Sham (N_pigs_ = 7, n_cells_ = 90-330/region).

**B,** Summary data of pooled regional cellular APD50 (left) and APD25 (right). Multilevel mixed model with Bonferroni correction.

## Figure S5


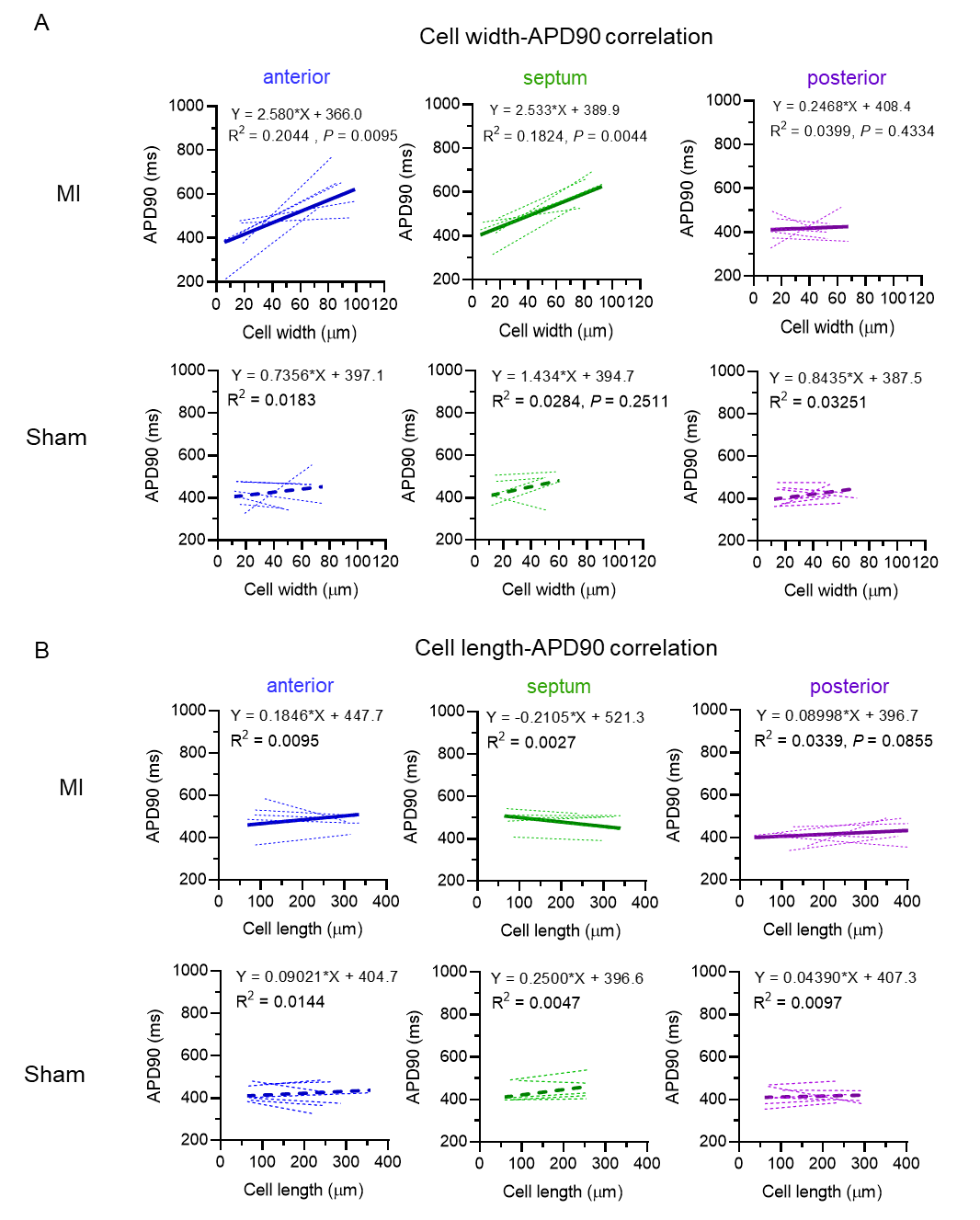


**Figure S5. Cell size correlation of action potential duration.**

**A,** Correlation between cell width and APD90 from MI (N_pigs_ = 6, n_cells_ = 86-216) and Sham (N_pigs_ = 7, n_cells_ = 90-330). Linear regression analysis.

**B,** Correlation between cell length and APD90 from MI, N_pigs_ = 6, n_cells_ = 86-216 (top) and Sham, N_pigs_ = 7, n_cells_ = 90-330 (bottom). Linear regression analysis.

## Figure S6


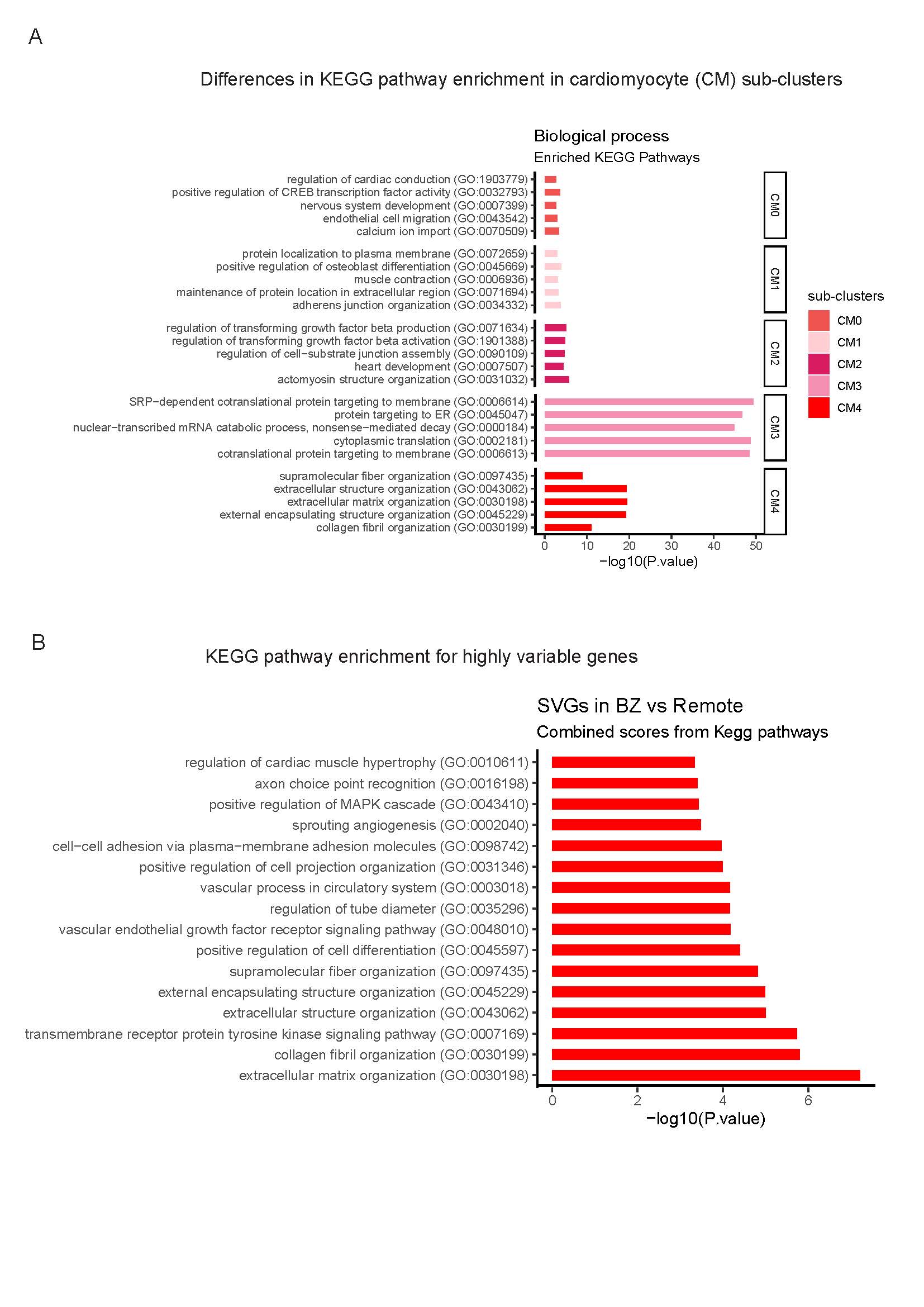


**Figure S6. Gene expression analysis of BZ vs. Remote cardiac myocytes.**

**A,** GO and KEGG pathway analysis characterizing cardiomyocyte (CM) sub-clusters.

**B,** GO and KEGG pathway analysis of the highly variable genes (detected by a Bayesian inference model) in BZ compared with Remote cardiomyocyte nuclei.

**Figure S7**


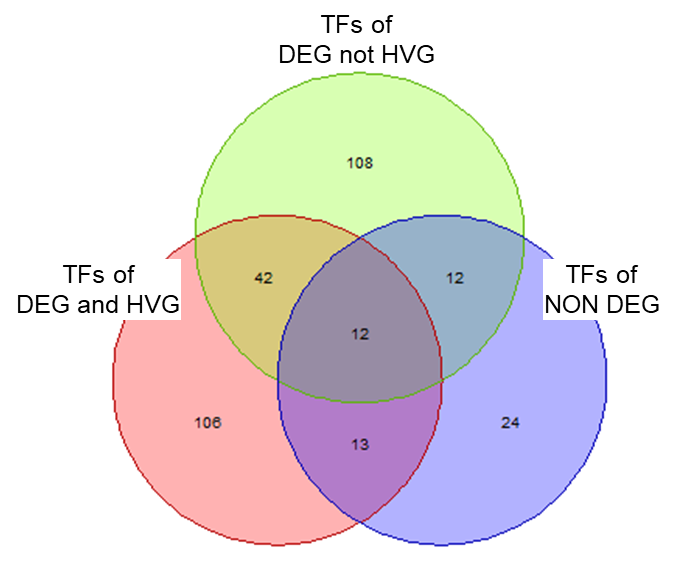


**Figure S7.Transcription factor analysis of BZ vs. Remote gene transcripts in cardiac myocytes.**

In the red circle are 173 TFs of DEGs that are also HVG. Removing the overlap with TFs that also regulate DEGs that are not HVG, and with TFs that also regulate non DEG, leaves 106 TFs that are specific for the HVG and DEGs: *ARID3A, NKX2-1, SPIC, TFCP2, TFAP2A, TFAP2B, TFAP2C, TEAD1, MYB, FOXA1, FOXA2, FOXA3, NFIA, NFIC,* ***EBF1****, PHTF1, TRIM28, NKX2-5, FOXN4, NR2E1, ZSCAN4-PS1, ZSCAN4-PS2, ZSCAN4-PS3, ZSCAN4B, ZSCAN4C, ZSCAN4D, ZSCAN4E, ZSCAN4F, STAT3, E2F6, PAX5, GTF2IRD1, PROX1, AHR, ARNT, TLX3, PARP1, ZFP384, ZBTB33, ZFP329, GATA1, GATA3,* ***GATA6****, ZFP217, IKZF2, SIX1, SIX2, GM10770, ZFP667, ZFP692, SMAD4, ZFP57, HES7, MED30, SPZ1, ZFP324, METTL3, SMARCA1, SOX30, PEG3, POU2F2, NR4A1, SOX1, ILF2, NFATC2, FOXB1, IRF7, SALL4, HOXD10, CLOCK, MEIS1, RUNX3, CEBPB, STAT6, ZFP3, FOXC1, FOXC2, FOXS1, SALL3, 1700009N14RIK, RAN, USF1, IRF2, FOXI1, ESRRB, IRF1, FOXG1, ZSCAN21, POU4F1, E4F1, GCM1, HES1, EGR4, YOD1, HOXD9, CEBPA, CEBPD, CEBPE, CEBPG, NR3C1, ZFP74, GSX2, TBP, POU2F1, HOXA10, TBX3*

From these 106 TFs, only ***GATA6*** is itself upregulated in BZ vs Remote.

GATA motifs are found in the *NPPB* and *SCN3B* promoters, which are both DEG and HVG and upregulated in the BZ.

From these 106 TFs, only ***EBF1*** is itself a HVG.

## Figure S8


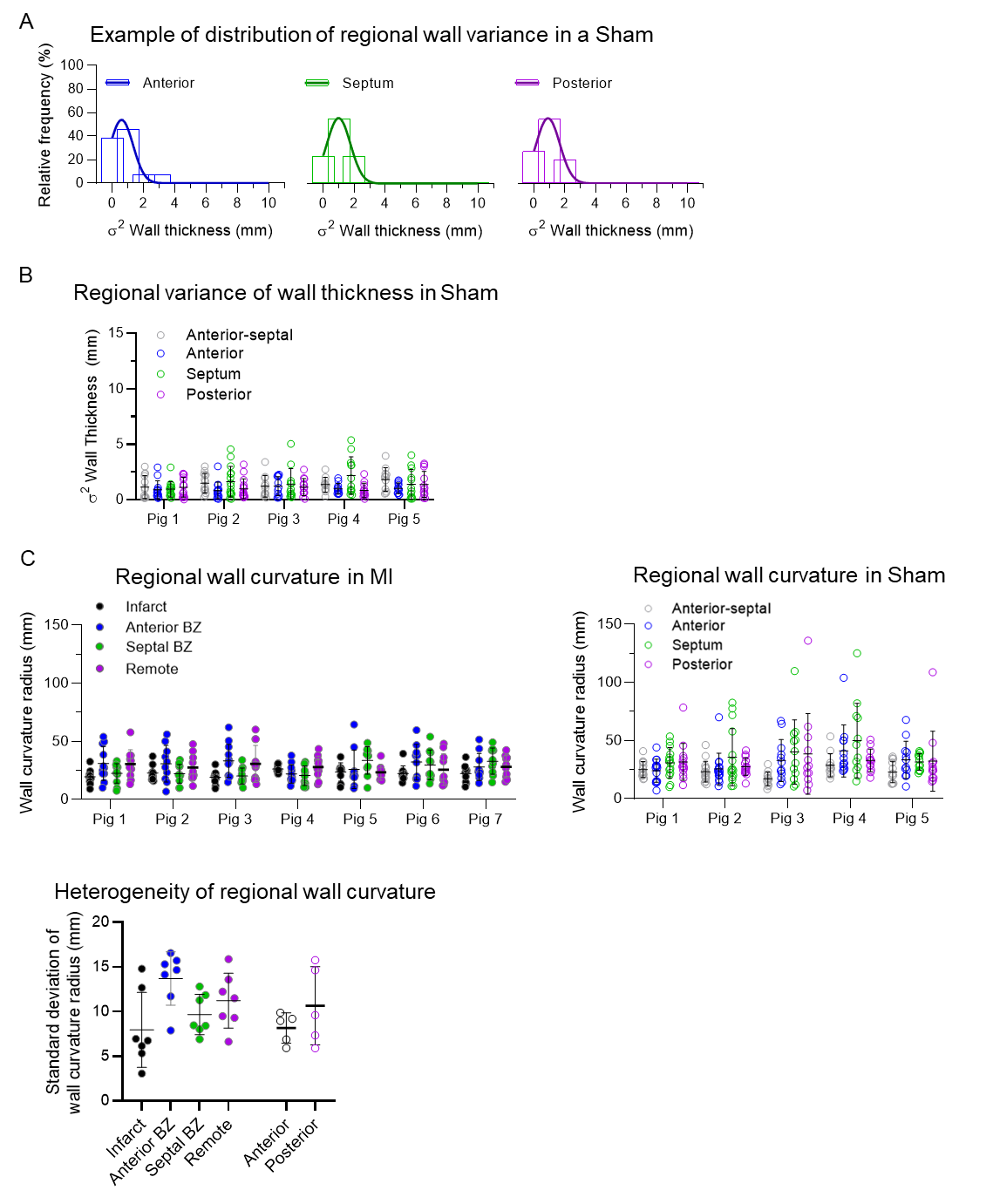


**Figure S8. Regional wall properties from MRI image analysis.**

**A,** Example of the distribution of regional variance (σ^2^) in wall thickness from Sham (n_slices_ = 10-13) illustrating uniformity in the different regions.
**B,** Regional wall variance (σ^2^) in vivo from each slice of the cMRI from Sham (N_pigs_ = 5, n_slices_ = 10-14).

**C,** Regional wall curvature in vivo from MI (left, N_pigs_ = 6, n_slices_ = 10-14) and Sham (N_pigs_ = 5, n_slices_ = 10-14).

**D,** Summary data of the heterogeneity of wall curvature, quantified by standard deviation from (C). Mixed model ANOVA with Bonferroni posttest.

# References

Refs 1-42 are included in the main manuscript

13. Dries E, Amoni M, Vandenberk B, Johnson DM, Gilbert G, Nagaraju CK, Puertas RD, Abdesselem M, Santiago DJ, Roderick HL, et al. Altered adrenergic response in myocytes bordering a chronic myocardial infarction underlies in vivo triggered activity and repolarization instability. *J Physiol*. 2020;598:2875–2895. doi: 10.1113/JP278839

15.Amoni M, Claus P, Dries E, Nagaraju C, De Buck S, Vandenberk B, Ingelaere S, Vermoortele D, Roderick HL, Sipido KR, et al. Discrete sites of frequent premature ventricular complexes cluster within the infarct border zone and coincide with high frequency of delayed afterdepolarizations under adrenergic stimulation. *Heart Rhythm*. 2021;18:1976–1987. doi: 10.1016/j.hrthm.2021.07.067

20.Litviňuková M, Talavera-López C, Maatz H, Reichart D, Worth CL, Lindberg EL, Kanda M, Polanski K, Heinig M, Lee M, et al. Cells of the adult human heart. *Nature*. 2020;588:466–472. doi: 10.1038/s41586-020-2797-4

26. Eling N, Richard AC, Richardson S, Marioni JC, Vallejos CA. Correcting the mean-variance dependency for differential variability testing using single-cell RNA sequencing data. *Cell Systems*. 2019;9:401–413. doi: 10.1016/j.cels.2019.08.003

43. Galan DT, Bito V, Claus P, Holemans P, Abi-Char J, Nagaraju CK, Dries E, Vermeulen K, Ventura-Clapier R, Sipido KR and Driesen RB. Reduced mitochondrial respiration in the ischemic as well as in the remote nonischemic region in postmyocardial infarction remodeling. *Am J Physiol Heart Circ Physiol*. 2016;311:H1075-H1090. doi: 10.1152/ajpheart.00945.2015

44. Leon DG, Lopez-Yunta M, Alfonso-Almazan JM, Marina-Breysse M, Quintanilla JG, Sanchez-Gonzalez J, Galan-Arriola C, Castro-Nunez F, Gonzalez-Ferrer JJ, Ibanez B, Perez-Villacastin J, Perez-Castellano N, Fuster V, Jalife J, Vazquez M, Aguado-Sierra J and Filgueiras-Rama D. Three-dimensional cardiac fibre disorganization as a novel parameter for ventricular arrhythmia stratification after myocardial infarction. *Europace.* 2019;21:822-832. doi: 10.1093/europace/euy306

45. Thienpont B, Aronsen JM, Robinson EL, Okkenhaug H, Loche E, Ferrini A, Brien P, Alkass K, Tomasso A, Agrawal A, Bergmann O, Sjaastad I, Reik W and Roderick HL. The H3K9 dimethyltransferases EHMT1/2 protect against pathological cardiac hypertrophy. *J Clin Invest*. 2017;127:335-348. doi: 10.1172/JCI88353

46. Preissl S, Schwaderer M, Raulf A, Hesse M, Gruning BA, Kobele C, Backofen R, Fleischmann BK, Hein L and Gilsbach R. Deciphering the Epigenetic Code of Cardiac Myocyte Transcription. *Circ Res*. 2015;117:413-23. doi: 10.1161/CIRCRESAHA.115.306337

47. Fleming SJ, Chaffin MD, Arduini A, Akkad A-D, Banks E, Marioni JC, et al. Unsupervised removal of systematic background noise from droplet-based single-cell experiments using CellBender. *Biorxiv* 2022:791699. doi:10.1101/791699.

48. Germain PL, Lun A, Garcia Meixide C, Macnair W and Robinson MD. Doublet identification in single-cell sequencing data using scDblFinder. *F1000Res*. 2021;10:979. doi: 10.12688/f1000research.73600.2.

49. Hao Y, Hao S, Andersen-Nissen E, Mauck WM, 3rd, Zheng S, Butler A, Lee MJ, Wilk AJ, Darby C, Zager M, Hoffman P, Stoeckius M, Papalexi E, Mimitou EP, Jain J, Srivastava A, Stuart T, Fleming LM, Yeung B, Rogers AJ, McElrath JM, Blish CA, Gottardo R, Smibert P and Satija R. Integrated analysis of multimodal single-cell data. *Cell*. 2021;184:3573-3587 e29. doi: 10.1016/j.cell.2021.04.048

50. Korsunsky I, Millard N, Fan J, Slowikowski K, Zhang F, Wei K, Baglaenko Y, Brenner M, Loh PR and Raychaudhuri S. Fast, sensitive and accurate integration of single-cell data with Harmony. *Nat Methods.* 2019;16:1289-1296. doi: 10.1038/s41592-019-0619-0

51. Bunis DG, Andrews J, Fragiadakis GK, Burt TD and Sirota M. dittoSeq: Universal User-Friendly Single-Cell and Bulk RNA Sequencing Visualization Toolkit. *Bioinformatics.* 2020;36:5535-6. doi: 10.1093/bioinformatics/btaa1011

52. Love MI, Huber W and Anders S. Moderated estimation of fold change and dispersion for RNA-seq data with DESeq2. *Genome Biol*. 2014;15:550. doi: 10.1186/s13059-014-0550-8

53. Kuleshov MV, Jones MR, Rouillard AD, Fernandez NF, Duan Q, Wang Z, Koplev S, Jenkins SL, Jagodnik KM, Lachmann A, McDermott MG, Monteiro CD, Gundersen GW and Ma'ayan A. Enrichr: a comprehensive gene set enrichment analysis web server 2016 update. *Nucleic Acids Res*. 2016;44:W90-7. doi: 10.1093/nar/gkw377

54. Blighe K, Rana S and Lewis M. EnhancedVolcano: Publication-ready volcano plots with enhanced colouring and labeling. GitHub, Inc. 2021.

55. Wickham H. ggplot2: Elegant Graphics for Data Analysis. New York, NY: Springer New York, NY. 2016.

56. Vallejos CA, Marioni JC and Richardson S. BASiCS: Bayesian Analysis of Single-Cell Sequencing Data. *PLoS Comput Biol*. 2015;11:e1004333. doi: 10.1371/journal.pcbi.1004333

57. Vallejos CA, Richardson S and Marioni JC. Beyond comparisons of means: understanding changes in gene expression at the single-cell level. *Genome Biol*. 2016;17:70. doi: 10.1186/s13059-016-0930-3
